# Lipidomic risk score independently and cost-effectively predicts risk of future type 2 diabetes: results from diverse cohorts

**DOI:** 10.1186/s12944-016-0234-3

**Published:** 2016-04-04

**Authors:** Manju Mamtani, Hemant Kulkarni, Gerard Wong, Jacquelyn M. Weir, Christopher K. Barlow, Thomas D. Dyer, Laura Almasy, Michael C. Mahaney, Anthony G. Comuzzie, David C. Glahn, Dianna J. Magliano, Paul Zimmet, Jonathan Shaw, Sarah Williams-Blangero, Ravindranath Duggirala, John Blangero, Peter J. Meikle, Joanne E. Curran

**Affiliations:** South Texas Diabetes and Obesity Institute, University of Texas Rio Grande Valley School of Medicine, Brownsville, TX 78520 USA; Baker IDI Heart and Diabetes Institute, Melbourne, VIC Australia; Department of Genetics, Texas Biomedical Research Institute, San Antonio, TX USA; Department of Psychiatry, Yale University School of Medicine, New Haven, CT USA; Olin Neuropsychiatry Research Center, Institute of Living, Hartford Hospital, 200 Retreat Avenue, New Haven, CT USA

**Keywords:** Diabetes, Endocrine disorders, Lipidomics, Diagnostic tools, Genetics

## Abstract

**Background:**

Detection of type 2 diabetes (T2D) is routinely based on the presence of dysglycemia. Although disturbed lipid metabolism is a hallmark of T2D, the potential of plasma lipidomics as a biomarker of future T2D is unknown. Our objective was to develop and validate a plasma lipidomic risk score (LRS) as a biomarker of future type 2 diabetes and to evaluate its cost-effectiveness for T2D screening.

**Methods:**

Plasma LRS, based on significantly associated lipid species from an array of 319 lipid species, was developed in a cohort of initially T2D-free individuals from the San Antonio Family Heart Study (SAFHS). The LRS derived from SAFHS as well as its recalibrated version were validated in an independent cohort from Australia – the AusDiab cohort. The participants were T2D-free at baseline and followed for 9197 person-years in the SAFHS cohort (*n* = 771) and 5930 person-years in the AusDiab cohort (*n* = 644). Statistically and clinically improved T2D prediction was evaluated with established statistical parameters in both cohorts. Modeling studies were conducted to determine whether the use of LRS would be cost-effective for T2D screening. The main outcome measures included accuracy and incremental value of the LRS over routinely used clinical predictors of T2D risk; validation of these results in an independent cohort and cost-effectiveness of including LRS in screening/intervention programs for T2D.

**Results:**

The LRS was based on plasma concentration of dihydroceramide 18:0, lysoalkylphosphatidylcholine 22:1 and triacyglycerol 16:0/18:0/18:1. The score predicted future T2D independently of prediabetes with an accuracy of 76 %. Even in the subset of initially euglycemic individuals, the LRS improved T2D prediction. In the AusDiab cohort, the LRS continued to predict T2D significantly and independently. When combined with risk-stratification methods currently used in clinical practice, the LRS significantly improved the model fit (*p* < 0.001), information content (*p* < 0.001), discrimination (*p* < 0.001) and reclassification (*p* < 0.001) in both cohorts. Modeling studies demonstrated that LRS-based risk-stratification combined with metformin supplementation for high-risk individuals was the most cost-effective strategy for T2D prevention.

**Conclusions:**

Considering the novelty, incremental value and cost-effectiveness of LRS it should be used for risk-stratification of future T2D.

**Electronic supplementary material:**

The online version of this article (doi:10.1186/s12944-016-0234-3) contains supplementary material, which is available to authorized users.

## Background

Type 2 diabetes (T2D) represents a complex metabolic turmoil rather than just a derangement of blood glucose control. In the continued quest for biomarkers of T2D [1] methods that can tap the whole gamut of metabolic disruption are therefore urgently required. In this context, there is a growing realization of the existence of the “plasma lipidome” that encompasses the entire spectrum of circulating lipid molecules [2, 3]. New high-throughput and high-resolution technologies applied to plasma lipidomic profiling are now providing additional insights into disease pathophysiology and individual lipid species have been associated with existing T2D or prediabetes [4–7]. However, the potential use of plasma lipid species as biomarkers to predict future development of T2D remains unexplored.

In this study, we used rich phenotypic and lipidomic data from two large, well-characterized, population-based cohorts and investigated the potential of plasma lipid species as independent biomarkers of incident T2D. We derived a lipidomic risk score (LRS) based on significant association of plasma lipid species with time to T2D in initially T2D-free Mexican American participants (*n* = 771, see Additional file 1: Figures S1, S2 and Tables S1, S2) from the San Antonio Family Heart Study (SAFHS) [8–10]. After establishing the independent and additive value of this LRS in the SAFHS cohort, we validated these results in a geo-epidemiologically distinct cohort from Australia – the AusDiab cohort (*n* = 644, see Additional file 1: Figure S3 and Table S2) [7, 11].

## Methods

### Cohorts

Studies related to the development and accuracy of the LRS were conducted in the SAFHS cohort [8–10]. At baseline, this cohort consisted of 1,431 individuals from 42 large and extended pedigrees of Mexican Americans residing in San Antonio, Texas, USA. Our protocol for selecting 771 individuals without diabetes who were followed for up to three additional visits spaced approximately 5 years apart (9197.1 person-years of follow-up with a maximum follow-up of 23.53 years) is shown in (Additional file 1: Figure S1). The distribution of SAFHS participants across families, their familial relationships and clinical characteristics and are shown in, (Additional file 1: Figure S2, Table S1 and Table S2). To determine the generalizability of the LRS derived from the SAFHS data, we used an independent well-characterized, high-risk subset of predominant Europid origin individuals drawn from the AusDiab cohort [7, 11]. From this cohort, we selected 653 participants who were initially non-diabetic. Since follow-up information was not available on 9 (1.4 %) individuals we included 644 participants in association analyses who were followed up for 5929.5 person-years (maximum follow-up 12.94 years). The algorithm used for inclusion of AusDiab participants is given in Additional file 1: Figure S3 and their clinical characteristics are shown in (Additional file 1: Table S2).

### Outcomes

Our primary outcome of interest was incident T2D, defined as T2D detected during follow-up visits in participants who did not have T2D at the baseline visit. At each visit, T2D was considered present when one or both of the following conditions were met: i) fasting plasma glucose ≥126 mg/dl (7.0 mmol/l) or 2-hour post-glucose load ≥200 mg/dl (11.1 mmol/l)]; ii) receipt of anti-diabetic medication. There were 122 (15.8) and 233 (36.2 %) cases of incident T2D in the SAFHS and AusDiab cohorts, respectively.

We also examined the association of the LRS with six measures of insulin resistance. These measures were [12]: fasting plasma glucose (FPG), fasting plasma insulin (FPI), homeostatic model of assessment – insulin resistance (HOMA-IR), quantitative insulin sensitivity check index (QUICKI) [13], McAuley index [14] and leptin/adiponectin ratio [15]. HOMA-IR was defined as FPG (mg/dl) x FPI (IU/L)/405; QUICKI was defined as 1/(log FPI + log FPG (mg/dl)) and McAuley’s index was calculated as exp [2.63 – 0.28 ln(FPI) – 0.31 ln(serum triglycerides in mmol/l)]. The methods used for measurement of FPG, FPI, leptin and adiponectin have been previously described [8–10, 16, 17].

### Lipidomic studies

Lipidomic profiling was conducted in the Metabolomics Laboratory, Baker IDI Heart and Diabetes Institute, Australia. We quantified a total of 319 lipid species (descriptive statistics are in, Additional file 1: Table S3) in plasma using a combination of liquid chromatography and electrospray ionisation-tandem mass spectrometry.

These methods have been described extensively elsewhere [7, 18–22]. Briefly, 10 μL aliquots of plasma were combined with 200 μL CHCl_3_/MeOH (2:1) and 15 μL of internal standard mix. Then samples were mixed (rotary mixer, 10 min), sonicated (water bath, 30 min) and allowed to stand (20 min) at room temperature. Afterwards, the samples were centrifuged (16,000xg, 10 min) and the supernatant was dried under a stream of nitrogen at 40 °C. Extracted lipids were resuspended in 50 μL H_2_O saturated BuOH, followed by 50 μL of 10 mM NH_4_CHOO in MeOH. Extracts were centrifuged (3,350xg, 5 min) and the supernatant transferred into 0.2 mL glass inserts in vials with teflon lined caps. Lipid measurements were performed by liquid chromatography electrospray ionisation-tandem mass spectrometry using an Applied Biosystems 4000 QTRAP. Liquid chromatography was performed on a Zorbax C18, 1.8 μm, 50 × 2.1 mm column at 300 μL/min using the following gradient conditions; 0 % B to 100 % B over 8.0 min, 2.5 min at 100 % B, a return to 0 % B over 0.5 min then 3.0 min at 0 % B prior to the next injection. Diacylglycerols and triacylglycerols were separated using the same solvent system with an isocratic flow (100 μL/min) of 85 % B over six minutes. Solvent A and B consisted of tetrahydrofuran:methanol:water in the ratios (30:20:50) and (75:20:5) respectively, both containing 10 mM NH_4_COOH. Quantification of individual lipid species was then performed using scheduled multiple-reaction monitoring (MRM) in positive ion mode [21, 22]. Lipid concentrations were calculated by relating the peak area of each species to the peak area of the corresponding internal standard. Cholesteryl ester species were corrected for response factors determined for each species.

### Statistical analysis

#### Data normalization

To ensure that the lipidomic traits are normally distributed and represented on a comparable metric, we transformed the raw plasma concentration of each lipid species by inverse-normalization that included ranking, generation of cumulative density functions and determination of z-values based on the cumulative density.

#### Mixed effects Cox proportional hazards modeling

In the SAFHS cohort, we used mixed effects Cox Proportional Hazards models that account for the fixed effects of the predictors as well as random effects due to kinship [23, 24]. We created a kinship matrix representing paired genetic relationships among study subjects. The elements of a kinship matrix (φ) indicate the genetic similarity (kinship coefficient) denoted by relationships between each pair of the study subjects. For example, the routinely used kinship coefficients for different relationships are as follows: identical twins, 1; parent-offspring or sibling, 0.5; and grandparent-grandchild, avuncular, half-siblings or double first cousins, 0.25. Further, the kinship coefficients for 3rd, 4th, 5th and 6th degree relatives are 0.0078, 0.0020, 0.0005 and 0.0001, respectively.

In these mixed effects Cox PH models we adjusted the association of lipid species with time to T2D for the following covariates: age, age^2^, sex, age x sex interaction, age^2^ × sex interaction, systolic and diastolic blood pressures (SBP and DBP), waist circumference, body mass index (BMI), total serum cholesterol, serum high-density lipoprotein (HDL) cholesterol, serum triglycerides and use of anti-lipid and anti-hypertensive drugs. We corrected for the potentially false positive results using Benjamini and Hochberg’s [25] method of controlling the false discovery rate (FDR). All p-values were two-tailed.

#### Receiver operating characteristics curves for family data

Parallel to the reasoning for using mixed effects Cox PH analyses, we also accounted for kinship structure in the receiver operating characteristics (ROC) curves using a variance components approach. Receiver operating characteristic curves plot a series of estimated sensitivity (true positive rate) and 1-specificity (false positive rate) pairs when a continuous predictor is dichotomized at various cut-offs [26, 27]. In the context of family studies, the estimates of sensitivity and specificities can be biased due to the kinships. To account for the kinships in the estimation of sensitivity and specificity we used the variance components approach and polygenic regression modeling in the following way.

For a given cut-off, we used a liability threshold model [28] for analysis of discrete traits and estimated the prevalence of T2D events separately in participants above and below the cut-off. We used polygenic regression models for this which permitted accounting for both the kinships as random effects and the abovementioned clinical predictors as fixed effects. A polygenic regression model is of the form:

$$ LT = \mu +\boldsymbol{\beta} \boldsymbol{a}+{g}_i+{e}_i $$

where, *LT* is the liability threshold, *μ* is the overall mean *LT*, ***β*** is the regression coefficient vector corresponding to the covariate matrix ***a***, *g*_*i*_ is the polygenic effect (used to estimate the heritabilities) and *e*_*i*_ is the measurement error. The mean (*μ*) represents the cumulative distribution function, the inverse of which provides probability. In the case of discrete traits this probability represents the prevalence of a condition.

Since we estimated the prevalence estimates in subset of subjects who were above or below the cut-off for a predictor, these prevalence estimates represent the post-test probability of a positive (p1) and negative (p0) result. Since the proportion of subjects above the cut-off (p) can also be estimated from the sample (through a similar polygenic regression model); we derived the Bayesian estimates of sensitivity and specificity as follows: sensitivity = p*p1/[p*p1 + (1-p)*p0] and specificity = (1-p)*(1-p0)/[(1-p)*(1-p0) + p*(1-p1)].

We repeated this procedure over the entire spectrum of observed cut-off values and plotted the ROC curve as tuples of sensitivity and 1-specificity. These estimates implicitly account for the kinship structure of the study subjects. We then used the methods described by Hanley and McNeil [26] to determine the area under the ROC curve (AUC, a measure of the predictive accuracy) and its standard error. We used the chi-square tests based on AUCs and their standard errors [29] to test for significant difference between AUCs.

#### Incremental value of plasma LRS

We determined the incremental value of lipidomic biomarkers to commonly used methods of risk stratification with respect to the following five aspects – model fit (assessed by likelihood ratio *χ*^2^, LRχ^2^), information content (Akaike information criterion, AIC), accuracy (Uno’s survival C statistic [30]), discrimination (integrated discrimination improvement, IDI) and continuous version of reclassification (net reclassification index, NRI).

#### Validation studies in the AusDiab cohort

In the AusDiab cohort, we used Poisson regression models to account for length time bias (using length of follow-up as an exposure variable) since the exact date of T2D diagnosis was unknown. We took three complementary approaches for validation of the LRS: i) the LRS derived from SAFHS was directly applied to the AusDiab participants; ii) the LRS was recalibrated for the AusDiab cohort; and iii) the predictive performance of the recalibrated score in AusDiab was compared to a similar set of Poisson regression models in the SAFHS cohort. To increase the generalizability of these interpretations, the confidence intervals (CI) were estimated using a bootstrap procedure on 1000 replicates. We also estimated AIC, IDI and NRI to quantify the improved prediction due to LRS in the AusDiab cohort.

#### Cost-effectiveness studies

We investigated if the use of LRS - alone or in combination with other screening methods – would be a cost-effective option in the setting of T2D screening. For this we considered seven potentially useful screening and intervention strategies (Fig. 4a) and compared the cost and effectiveness of these strategies.

All the screening strategies considered in these analyses assume:

1. Single payer perspective
2. A one-time screening with the indicated strategy;
3. Identification of differential risk groups (high risk, moderate risk or low risk) based on the strategy used;
4. Influence of the screening/interventions strategy on the 5-year observed probability of incident T2D;
5. A willingness-to-pay (WTP) US$ 4450.12 for a 5-year T2D prevention program. This estimate is based on the 3-year estimates of WTP reported by Johnson et al. [31], linearly extrapolated to five years and converted to 2015 US$; and
6. Even though the probability estimates used here are derived from both SAFHS and AusDiab, the analyses assume US target population to which the cost-effectiveness measures apply.

##### Cost estimations

For estimating the cost of interventions, we used the 10-year cost-effectiveness data reported by the DPP Trial [32, 33]. We cumulated the first five years data from the DPP Trial in individuals undergoing Lifestyle intervention, Metformin supplementation or No intervention (placebo) group. We also considered the medical treatment costs for individuals with and without T2D outside the DPP. We then converted these costs into 2015 US$ which represent the undiscounted costs from a single-payer’s perspective. It is recommended that the costs and QALYs be discounted for future outcomes in studies of prevention strategies such as ours. However, the rate at which the costs and QALYs should be discounted (currently recommended to be 3.5 % for both [34]) is a subject of an ongoing debate [35–38]. In this study, we chose to use undiscounted costs and QALYs since our aim was to conduct proof-of-principle cost-effectiveness analyses rather than to provide a futuristic solution to screening for T2D. Details of the cost estimations are shown in (Additional file 1: Table S7). To estimate the costs of screening, we used different sources of data. For the costs associated with fasting plasma glucose (FPG), we used the costs as mentioned by Sullivan et al. [39] and converted these costs into 2015 US$. To estimate the cost of risk factor screening using a questionnaire method, we used data as reported by Zhang et al. [40] and converted these costs into 2015 US$. The cost of the LRS was established as follows: assuming a center that conducts a large number of lipidomic assays, the cost of each lipidomic species measured is ~1 US$ (total 3 US$ for three lipid species), the cost of mass spectrometry is ~4 US$ and the cost of manpower for the assay is 4 US$ totaling to US$11 per assay. For screening strategies that used only LRS, there would be an additional cost of plasma sample preparation (29 US$, based on costs reported at: http://pathology.med.wayne.edu/lipidomics/servicescosts.php) and a primary care visit (50 US$, reported in [40]). However when the LRS is combined with the fasting plasma glucose assays then these additional costs of sample preparation and primary care visit are charged only once and not duplicated for each assay separately.

##### Effectiveness measures

We used two measures of effectiveness – risk reduction in the incidence of T2D and quality-adjusted life years (QALYs). The estimates of QALY were derived from the DPP data which report the annual QALYs stratified by T2D status and intervention received. Risk reduction in the incidence of T2D was estimated as a function of the probability of T2D at five years and the expected probability based on efficacy of the intervention used. We used an estimate of 0.58 and 0.31 as efficacy of lifestyle intervention and metformin supplementation (500 mg once daily), respectively, based on the DPP Trial results [33, 41].

##### Outcomes of cost-effectiveness studies

These were: cost and effectiveness, incremental cost-effectiveness ratios (ICER) and net monetary benefits. Results were represented as cost-effectiveness plots, tornado diagrams and ICER plots.

##### Analytical approach for cost-effectiveness studies

We conducted cost-effectiveness studies separately for the SAHFS and AusDiab datasets. For each dataset, we used the same estimates of costs and effectiveness measures (as detailed above). The probability of being in either the high risk (HR), moderate risk (MR) or low risk (LR) was different for the two study cohorts; hence two different sets of analyses were used. For each dataset, we first studied the distribution of the risk groups and then conducted cost-effectiveness studies by i) rolling back the decision tree shown in (Additional file 1: Figure S6); and ii) plotting the cost and effectiveness. Since there were two effectiveness measures, we combined these measures into a single plot by using ordinate axis for QALYs and altering the size of marker based on the risk reduction in T2D incidence (Fig. 4b and c). We then estimated the ICERs based on estimated cost and QALYs for each strategy (see Additional file 1: Table S8). These analyses together represented the base case scenarios. Finally, we conducted sensitivity analyses by first using tornado plots (see Additional file 1: Figure S7) and then conducting a one-way sensitivity analysis for the variable to which the expected value (EV) was most sensitive. For the one-way sensitivity analyses, we used microsimulation with 1000 runs and the results were expressed as mean cost/QALY ratio for each strategy based on the 1000 runs. Further, we smoothed the results using fourth order polynomial regression analyses to capture the potential non-linearity in the association of EV with the sensitivity variable.

#### Statistical software

We used the following statistical programs (with the analytical purpose): Sequential Oligogenic Linkage Analysis Routines – SOLAR [42] (inverse normalization of traits, polygenic regression models, ROC); coxme in R (mixed effects Cox PH models, LRχ^2^, AIC); survC1 [43] in R (Uno’s survival C statistic); survIDINRI [44] in R (IDI and NRI estimation), Stata 12.0 (Stata Corp, College Station, TX; data management, FDR corrected p-values, Poisson regression analyses, improved prediction indices in the AusDiab cohort and Cuzick’s nonparametric test for linear trend), and TreeAge Pro 2015 (TreeAge Software, Inc, Williamstown, MA; cost-effectiveness analyses).

## Results

### Development of LRS in the SAFHS cohort

**Fig. 1:**
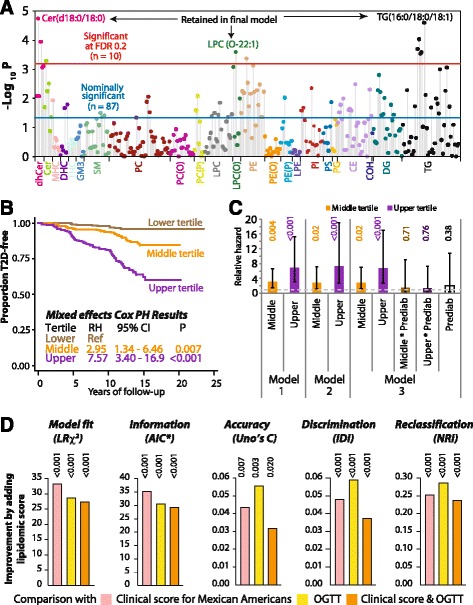
Development and assessment of the LRS in the SAFHS cohort. **a** Manhattan plot showing the association of the entire lipidome with incident T2D. All results are from mixed effects Cox proportional hazards models that account for degree of relationship among participants. All models are adjusted for age, age^2^, sex, age x sex interaction, age^2^ × sex interaction, systolic and diastolic blood pressures, waist circumference, body mass index, total serum cholesterol, serum high-density lipoprotein cholesterol, serum triglycerides and use of anti-lipid and anti-hypertensive drugs. The dots are color-coded for different lipid classes. The blue horizontal line represents a nominal type I error rate of 0.05 while the red horizontal line represents a global, FDR-corrected type I error rate of 0.2. All lipid species above the red line were simultaneously included in stepwise regression models and the species that were retained in the final multivariate model are indicated at the top of the plot. **b** Association of the LRS with incident T2D. Based on the regression coefficients from this final model, we derived a LRS as 0 · 4176*i(Cer(d18:0/18:0))-0 · 3443*i(LPC(O-22:1)) + 0 · 5361*i(TG(16:0_18:0_18:1)) where i(L) represents the inverse-normalized plasma concentration of lipid species L. The individuals were classified into three groups based on tertiles of the LRS. Shown is a Kaplan-Meier plot of progression to T2D based on the LRS. The results of mixed effects Cox proportional hazards models are after adjusting for the same covariates as those listed in Panel A. RH, relative hazard. **c** Association of the LRS with incident T2D independent of prediabetes. The plot shows relative hazard (colored bars), 95 % confidence intervals (error bars) and p-values (rotated numbers above bars). Model 1 – stratified mixed effects Cox PH model with presence of prediabetes as the stratifying variable; Model 2 – mixed effects Cox PH model restricted to NGT participants only; Model 3 – Interactive model that included interaction terms between presence of prediabetes and the LRS tertiles. All models use participants in the lower tertile of LRS as the reference category. NGT, normal glucose tolerance; Prediab, presence of prediabetes. **d** Clinical value of the LRS as a biomarker of incident T2D. Plots show bars representing improvement in the indicated characteristic achieved by adding LRS to the color coded models. Three models were used: clinical score for Mexican Americans (pink bars), oral glucose tolerance test (yellow bars) and clinical score combined with oral glucose tolerance test (orange bars). Statistical significance of the improvement is shown by the rotated numbers above the bars. *, the plot shows decrease in this parameter as a measure of improvement; LRχ^2^, likelihood ratio *χ* ^2^ statistic; AIC, Akaike information criterion; Uno’s C, Uno’s C statistic for survival data; IDI, integrated discrimination improvement; NRI, continuous net reclassification index

In the SAFHS cohort, there was a wide inter-individual variability in plasma concentrations of the lipid species (see Additional file 1: Table S3). Thus, we transformed these plasma concentrations using an inverse-normalization procedure for use as independent variables in mixed effects Cox regression modeling. The results demonstrated that after accounting for clinically relevant covariates, 87 of the 319 lipid species predicted T2D with a nominal *p*-value <0 · 05 but only 10 lipid species had a false discovery rate (FDR) corrected p-value <0 · 2 (Fig. 1a, see Additional file 1: Table S4). We then included these 10 species (see Additional file 1: Figure S4A) in backward-elimination stepwise regression models and found that the final model retained only three lipid species (Fig. 1a, see Additional file 1: Figure S4A): dihydroceramide 18:0 (Cer(d18:0/18:0)), lysoalkylphosphatidylcholine 22:1 (LPC(O-22:1)) and triacylglycerol 16:0/18:0/18:1 (TG(16:0/18:0/18:1)). LPC(O-22:1) was associated with a slower disease onset while the other two species were associated with faster progression to T2D. From these results we generated a composite LRS as shown in (Additional file 1: Figure S4B). To compare the predictive accuracy of the lipid score against each of its components, we conducted ROC analyses (see Additional file 1: Figure S4C) which showed that the predictive accuracy of the composite LRS (AUC 0 · 7566, 95 % CI 0 · 7111 – 0 · 8021) was significantly better than that of any component species.

### Prediction of future T2D based on LRS in the SAFHS cohort

We stratified the SAFHS participants into tertiles of the LRS. Participants in the middle and upper tertile groups of the LRS progressed ~3 and ~8 times faster to T2D, respectively, as compared to those in the lower tertile group (Fig. 1b) even after accounting for all the relevant clinical and biochemical confounders. Corroborating these observations we found that by 15 years of follow up 3 · 5, 12 · 5 and 30 · 7 % of the participants in the lower, middle and upper tertile groups had progressed to T2D, respectively (see Additional file 1: Figure S4D). Since prediabetes [defined as the presence of impaired fasting glucose (IFG) and/or impaired glucose tolerance (IGT)] was a strong determinant of incident T2D risk in the SAFHS participants (see Additional file 1: Figure S5), we investigated if our results of lipidomic associations were confounded by prediabetes. For this, we ran three complementary regression models – a stratified model (prediabetes as the stratifying variable), a model restricted to participants with normal glucose tolerance (NGT) and an interactive model that included interaction between the LRS tertiles and prediabetes. Our results (Fig. 1c) showed that irrespective of the modeling approach used, the participants in the middle and upper tertile of the LRS progressed significantly faster to T2D as compared to the participants in the lower tertile.

Corroborating these results, we observed that the median LRS scores showed a clear gradient in association with the T2D status (see Additional file 1: Table S5). Individuals, who were NGT at baseline and remained NGT throughout follow-up had the lowest median score, while those were prediabetic at baseline but did not develop T2D during follow-up had a higher median LRS (0.38 versus −0.24). The highest median LRS, however, was observed for individuals who developed T2D during follow-up (0.78). Cuzick’s test for linear trend showed that these median LRS values were linearly related to the T2D status and highly significant (*p* < 1.0x10^−22^).

### LRS-based improvement in clinical prediction of T2D in the SAFHS cohort

We then examined if the addition of the LRS to commonly used clinical predictors of T2D risk can significantly augment the predictive performance. We considered two sets of commonly used predictors: a clinical score tailored for Mexican Americans [45] and the oral glucose tolerance test (OGTT). The clinical score is based on age, sex, fasting glucose, systolic blood pressure, HDL cholesterol, BMI and family history of T2D [45]. Figure 1d shows that addition of the LRS to the clinical score and to the OGTT, singly or in combination, resulted in an improved model fit (higher likelihood *χ*^2^), information content (lower Akaike Informatin Criterion, AIC) and predictive accuracy (increased c-statistic of Uno). Integrated discrimination improvement (IDI) showed a 3 · 7 % – 5 · 9 % improvement in discrimination and the continuous net reclassification index (NRI) indicated an improved reclassification by addition of the LRS. The observed improvement in all indexes was statistically significant (Fig. 1d).

### Association of LRS with insulin resistance

**Fig. 2:**
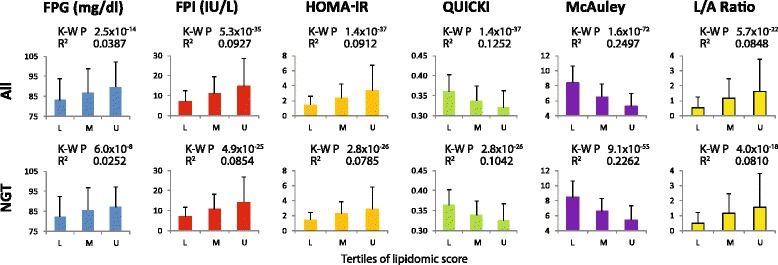
Association of LRS with measures of insulin resistance (IR) in SAFHS participants. Each plot shows the median (colored bars) and inter-quartile range (error bars) for the indicated measure of IR across tertiles of LRS. L, M and U represent lower, middle and upper tertiles of the LRS, respectively. K-W P, Kruskall-Wallis test significance value; R^2^, variability in the measure of IR explained by tertiles of LRS estimated using quantile regression; FPG, fasting plasma glucose; FPI, fasting plasma insulin; HOMA-IR, homeostatic model of assessment – insulin resistance; QUICKI, quantitative insulin sensitivity check index; L/A Ratio, leptin/adiponectin ratio; NGT, normal glucose tolerance

Mechanistically, insulin resistance (IR) provides an initial trigger to the pathogenesis of T2D [46–48]. Given the fact that the LRS predicted progression to T2D even in the NGT individuals, we considered whether the LRS was associated with IR in the SAFHS individuals. In the absence of data on the diagnostic euglycemic clamp, we used six previously established measures of IR [12]: fasting plasma glucose (FPG), fasting plasma insulin (FPI), homeostatic model of assessment – insulin resistance (HOMA-IR), quantitative insulin sensitivity check index (QUICKI) [13], McAuley index [14] and leptin/adiponectin ratio [15]. All the six indices of IR showed a consistently linear association with the LRS tertiles in all as well as in NGT individuals (Fig. 2). These findings afford a strong support to the hypothesis that the LRS may be detecting subclinical insulin resistance.

### Validation of LRS in the AusDiab cohort

**Fig. 3:**
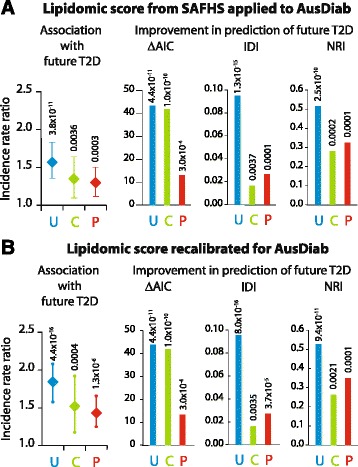
Validation of the LRS in the AusDiab cohort. **a** Direct application of the LRS derived from SAFHS cohort to the participants from AusDiab cohort. Left panel shows incidence rate ratio for future T2D associated with one standard deviation change in the LRS. The results are from three Poisson regression models that used duration of follow-up as an exposure variable: U – unadjusted; C – adjusted for clinical covariates; P – adjusted for presence of prediabetes. The clinical covariates used for adjustment in the multivariate models were: age, sex, systolic and diastolic blood pressure, body mass index, total cholesterol, HDL cholesterol, serum triglycerides, family history of diabetes, and use of anti-hypertensive and lipid-lowering drugs. The Right Panel shows three bar charts each of which depicts improvement in the indicated parameter upon addition of the LRS to the indicated regression model. Rotated numbers at the top of the bars are p-values. ΔAIC, improvement in Akaike Information Criterion; IDI, integrated discrimination improvement; NRI, continuous net reclassification index. **b** LRS recalibrated for AusDiab. Based on the results of Poisson regression analyses in the AusDiab, a recalibrated LRS was calculated as follows: 0.2259*i(Cer(d18:0/18:0))-0.2397*i(LPC 22:1) + 0.3267*i(TG(16:0_18:0_18:1)) where i(L) represents the inverse-normalized plasma concentration of lipid species L. Key to the plots in (Panel **b**) is the same for those in (Panel **a**)

Next, we validated the LRS in another independent cohort, the AusDiab cohort. The participants in the AusDiab cohort were on an average ~20 years older, were equally distributed across sex, had lower BMI but higher blood pressures, had higher total serum cholesterol and triglycerides and a higher prevalence of existing prediabetes at baseline as compared to the SAFHS participants (see Additional file 1: Table S2). Despite these differences, a direct validation of the LRS derived from the SAFHS showed that the score was associated with an increased risk of incident T2D in AusDiab participants even after accounting for clinical covariates or prediabetes at baseline (Fig. 3a, left panel). Since practical application of the LRS can include population-specific refinement, we recalibrated the LRS for the AusDiab cohort and again observed an increased risk of incident T2D (Fig. 3b, left panel). To examine the robustness of these findings we ran the analyses in 1000 bootstrap samples and found that the LRS continued to be a significant predictor of T2D in the AusDiab cohort. Similar to the observations in the SAFHS cohort, the AusDiab cohort also demonstrated a statistically significant linear trend in median LRS based on T2D status (see Additional file 1: Table S5). Moreover, to permit a direct comparison of this recalibrated score in the AusDiab cohort, we also ran corresponding mixed effects Poisson regression models in the SAFHS participants (see Additional file 1: Table S6) and observed that the LRS indeed provided excellent corroborative results in both cohorts. Further, we found that in all the modeling approaches taken in the AusDiab cohort, addition of the LRS (whether derived from SAFHS or recalibrated for the AusDiab cohort) was always associated with a statistically significant improvement in the information content, discrimination and reclassification (Fig. 3a and b, Right panels). Together, these results demonstrate that the LRS derived from the SAFHS participants was generalizable to an independent and distinct cohort.

### Cost-effectiveness modeling of LRS based screening and intervention strategies

**Fig. 4:**
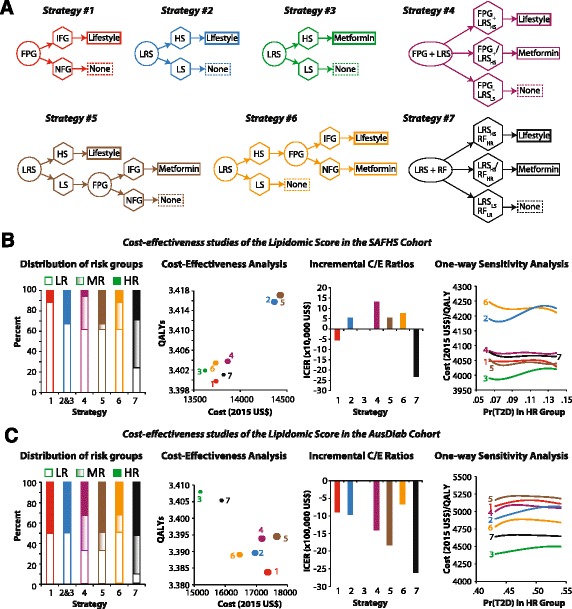
Cost effectiveness analyses of candidate screening and intervention strategies for T2D risk-stratification. **a** The seven strategies that were considered. The diagrams use the following convention: circles, name of the screening test; hexagons, results of the screening test; rectangles, suggested intervention; thick border for rectangles, high-risk group; thin border for rectangles, moderate-risk group; dashed border for rectangles, low-risk group. The abbreviations used in the panel are as follows: FPG, fasting plasma glucose; IFG impaired fasting glucose; NFG, normal fasting glucose; LRS, lipidomic risk score; HS, high score; LS, low score; RF, risk factor assessment. For LRS, a high score was defined on the basis of cutoffs defined by receiver-operating-characteristic curves. The strategies are color coded and the colors are consistently used in panels B and C. The full decision tree based on the seven strategies is shown in (Additional file 1: Figure S6). Details of the costs and utilities are provided in Material and Methods, (see Additional file 1: Figure S6, Tables S7 and S8). **b** Cost-effectiveness analyses based on the LRS in the SAFHS cohort. Leftmost plot shows the relative distribution of the risk groups based on the screening strategy used (LR, low-risk group; MR, moderate-risk group; HR, high-risk group). The plot second from left shows costs (2015 US$) and quality-adjusted life years (QALYs) for each strategy. The size of the markers is proportional to the expected risk reduction in T2D incidence. The next plot shows incremental cost-effectiveness ratios using the most cost-effective strategy (#3) as the reference strategy. The rightmost plot shows smoothed results of sensitivity analyses that used 1000 microsimulation runs. The results were smoothed using fourth-order polynomial regression lines. Sensitivity analyses shown here are for the variable (probability of T2D at 5-years in HR individuals) to which the decision was most sensitive based on the tornado diagrams shown in (Additional file 1: Figure S7). **c** Cost-effectiveness analyses based on the LRS in the AusDiab cohort. Key to plots is the same as those for plots in (Panel **b**). Full numerical results of cost effectiveness analyses for both cohorts are provided in (Additional file 1: Table S9)

Considering these results in totality, we next investigated whether T2D risk-stratification based on the LRS would be an economically viable alternative to existing risk stratification strategies. It is being recognized that screening for T2D with FPG combined with lifestyle intervention in high-risk individuals may be cost-effective compared to no screening at al. [39, 49, 50] We therefore considered whether the LRS – alone or in combination with FPG or clinical risk factors – would be cost-effective as compared to FPG alone for outcomes measured at the end of five years. We used the probability estimates from SAFHS and AusDiab cohorts, cost and utility estimates from the DPP trial [33, 41] and screening costs from other published studies [39, 50–52]. Details of the probabilities, costs and utilities are provided in Materials and Methods, (see Additional file 1: Tables S7 and S8). We compared seven potentially useful screening-and-intervention strategies (Fig. 4a, Additional file 1: Figure S6) and found (Fig. 4b, Additional file 1: Table S9) that the strategy of risk stratification using the LRS followed by metformin treatment for the high-risk individuals would be the most cost-effective screening strategy. In both SAFHS and AusDiab, this strategy was associated with least costs (see Additional file 1: Table S9) and was more cost-effective than the strategy of FPG followed by lifestyle intervention (negative incremental QALYs in both cohorts, see Additional file 1: Table S9). Similarly, the strategy of risk factor stratification combined with the LRS was also associated with negative incremental effectiveness in both cohorts. On the other hand, the proportion of potential T2D cases prevented by the LRS/metformin strategy was the least as compared to other candidate strategies considered. Therefore, practical use of the screening-and-intervention policy will likely be a trade-off between attempting to increase QALYs versus targeting aggressive reduction in T2D incidence. Lastly, the decision in favor of LRS/metformin strategy was most sensitive to the probability of T2D at five years in the high risk individuals (see Additional file 1: Figure S7). However, over the entire range of sensitivity analyses, the strategy of LRS/metformin for high-risk individuals remained the most cost-effective solution in both cohorts (Fig. 4b and c).

## Discussion

Our study reports five novel findings: i) the plasma LRS independently, accurately and additively predicted incident T2D; ii) given the LRS could accurately risk-stratify even the NGT participants indicates that the plasma lipidomic changes may precede those in glucose metabolism; iii) these changes are associated with insulin resistance; iv) the LRS is generalizable as validated in a distinctly different cohort; and v) the LRS combined with metformin supplementation for high-risk individuals is the most cost-effective screening strategy. Of note, supportive evidence for the biological plausibility of the three significant lipid species as T2D biomarkers is plentiful. We have previously shown that the dihydroceramide/ceramide, triacyglycerol and phosphatidylcholine axes are important targets in T2D pathophysiology [7]. The enzyme ceramide synthase 1 (specifically involved in the biosynthesis of Cer(d18:0/18:0), [53]) is inversely associated with alterations in murine models of insulin resistance [54, 55]. The negative association between lysoalkylphosphatidylcholine species and T2D risk is in agreement with the beneficial associations of LPC(O) observed in studies of overfeeding [56] and insulin resistance [57]. Lastly, the TG(16:0/18:0/18:1) has been reported as a prominent triacylglycerol species that discriminated between cases and controls of diabetes in the Framingham Offspring Study participants [58].

Two important findings from our study should be highlighted. First, it is instructive that the majority of the significant lipid species enlisted in (Additional file 1: Figure S4A) contained palmitate or stearate moieties both of which are preferred substrates for stearoyl-CoA desaturase [59] (SCD) – an enzyme involved in converting saturated to unsaturated fatty acids. This observation points towards a possibility of the derangement of SCD pathway as a forerunner of T2D. Interestingly, the EPIC-InterAct study [60] also found that the group of saturated fatty acids comprising myristic acid, palmitic acid and stearic acid is associated with a significantly increased risk of T2D. Second, since there is a strong genetic basis to T2D it is conceivable that the LRS may share some genetic influences with T2D. Genetic studies in future need to establish putative overlaps that can partly explain the genetics of T2D. Such studies need to carefully dissect out the potential associations of genetic variants, epigenetic infrastructure and gene expression on T2D susceptibility through an altered lipidomic signature.

## Conclusions

The lipidomic risk score based on plasma concentration of three, non-redundant plasma lipid species is an independent and additive biomarker of incident T2D. In the context of biomarkers for cardiovascular risk, Hlatky et al. [61] have suggested that “a novel risk marker should be evaluated in several phases, including initial proof of concept, prospective validation in independent populations, documentation of incremental information when added to standard risk markers, assessment of effects on patient management and outcomes, and ultimately, cost-effectiveness.” Our study extends this concept to T2D risk-stratification and demonstrates strong evidence for plasma LRS as a biomarker for future T2D.The strengths of our study include the scale of lipidomic profiling, prospective evaluation, two well-characterized cohorts, replicability of the accurate and independent value of LRS, its clinical value as assessed by IDI and NRI, and the cost-effectiveness analyses. The global epidemic of T2D beckons a combination of preventive strategies aimed at its control [62]. Considering the immense costs of diabetes management, it is incumbent upon us to evaluate effective preventive strategies which will hinge heavily on accurate and early detection [1, 63]. In that vein and to that end, our study suggests that while dysglycemia remains a direct measure of T2D, other subtle metabolic changes may precede it and methods aimed at detecting these early signals can play a significant role in the prevention and control of T2D.

### Ethics approval and consent to participate

The Institutional Review Board of the University of Texas Health Science Center at San Antonio approved the SAFHS study and informed consent was obtained from all the study participants. The lipidomic analysis of the AusDiab study was approved by the Alfred Hospital, Ethics Committee, Project No: 104/10.

### Availability of data and materials

The data are available with JEC, JB and PJM and will be shared upon request.

## Additional files

Additional file 1: This file contains Supplementary **Figures S1-S7** and Supplementary **Tables S1-S9**. (DOCX 427 kb)

## Abbreviations

AIC: Akaike information criterion
AUC: area under curve
EV: expected value
FDR: false discovery rate
FPG: fasting plasma glucose
FPI: fasting plasma insulin
HOMA-IR: homeostatic model of assessment – insulin resistance
HR: high-risk
ICER: incremental cost-effectiveness ratio
IDI: integrated discrimination improvement
IFG: impaired fasting glucose
IGT: impaired glucose tolerance
LR: low risk
LRS: lipidomic risk score
MR: moderate risk
NRI: net reclassification index
OGTT: oral glucose tolerance test
QALY: quality-adjusted life year
QUICKI: quantitative insulin sensitivity check index
ROC: receiver operating characteristic curve
SAFHS: San Antonio Family Heart Study
T2D: Type 2 diabetes
WTP: willingness-to-pay
