# Supplementary material for: Lipidomic risk score independently and cost-effectively predicts risk of future type 2 diabetes: results from diverse cohorts
Source: Lipids Health Dis. 2016 Apr 4;15:67. doi: 10.1186/s12944-016-0234-3 (PMC4820916; doi:10.1186/s12944-016-0234-3)
Supplement: Additional file 1: — This file contains Supplementary Figures S1-S7 and Supplementary Tables S1-S9. (DOCX 427 kb) [file 12944_2016_234_MOESM1_ESM.docx]

**SUPPLEMENTARY MATERIALS**

Title: Lipidomic Risk Score Independently and Cost-Effectively Predicts Risk of Future Type 2 Diabetes: Results from Diverse Cohorts

**Authors:** Manju Mamtani^1,*^, Hemant Kulkarni^1^, Gerard Wong^2^, Jacquelyn M. Weir^2^, Christopher K. Barlow^2^, Thomas D. Dyer^1^, Laura Almasy^1^, Michael C. Mahaney^1^, Anthony G. Comuzzie^3^, David C. Glahn^4,5^, Dianna J. Magliano^2^, Paul Zimmet^2^, Jonathan Shaw^2^, Sarah Williams-Blangero^1^, Ravindranath Duggirala^1^, John Blangero^1^, Peter J. Meikle^2,†^, Joanne E. Curran^1,†^

**Affiliations:**

^1^South Texas Diabetes and Obesity Institute, University of Texas Rio Grande Valley School of Medicine, Brownsville, TX, USA

^2^Baker IDI Heart and Diabetes Institute, Melbourne VIC, Australia

^3^Department of Genetics, Texas Biomedical Research Institute, San Antonio TX, USA

^4^Department of Psychiatry, Yale University School of Medicine, New Haven, CT, USA

^5^Olin Neuropsychiatry Research Center, Institute of Living, Hartford Hospital, 200 Retreat Avenue, CT, USA

† These authors jointly coordinated this research study.

***Contact information for corresponding author:**

Manju Mamtani, MD

South Texas Diabetes and Obesity Institute,

University of Texas Rio Grande Valley School of Medicine,

Brownsville, TX, 78520

Phone: (956) 882 7511

Fax: (956) 882 6834

E-mail: [manju.mamtani@utrgv.edu](mailto:manju.mamtani@utrgv.edu)

**Table of Contents**

*Section Title Page*

**1 Supplementary Figures 4-11**

Supplementary Figure S1: Selection of participants from the SAFHS cohort 4

Supplementary Figure S2: Distribution of SAFHS participants

across families 5

Supplementary Figure S3: Selection of participants from the

AusDiab cohort 6

Supplementary Figure S4: Development and accuracy of LRS in the

SAFHS cohort 7-8

Supplementary Figure S5: Association of prediabetes with incident diabetes

in the SAFHS cohort 9

Supplementary Figure S6: Structure of the Decision Tree used to investigate the

cost-effectiveness of screening strategies for T2D 10

Supplementary Figure S7: Sensitivity analyses for cost-effectiveness of

T2D screening based on tornado diagrams 11

**2 Supplementary Tables 12-32**

Supplementary Table S1: Family relationships among pairs of

SAFHS participants 12

Supplementary Table S2: Baseline characteristics of cohorts 13

Supplementary Table S3: Descriptive statistics of the plasma lipidome in the

SAFHS participants 14-20

Supplementary Table S4: Results of mixed effects Cox proportional hazards for

the association of each lipid species with

onset of T2D 21-27

Supplementary Table S5: Median LRS based on T2D status 28

Supplementary Table S6: Incidence risk ratio for the association of LRS with

future type 2 diabetes in the SAFHS cohort

estimated using mixed effects Poisson regression 29

Supplementary Table S7: Probabilities used in base case and

sensitivity analyses 30

Supplementary Table S8: Costs and health utilities at the end of five years 31

Supplementary Table S9: Summary of the results of base case

cost-effectiveness analyses 32

**Supplementary Figure S1: Selection of participants from the SAFHS cohort**

From the original study size of 1431 recruited Mexican Americans from large, extended families in San Antonio, TX we included a total of 771 non-diabetic subjects on whom complete phenotypic data including plasma lipidomic profiling was available. The following figure shows how algorithmically the subjects were selected. Colored boxes indicate subjects who were excluded.

**Supplementary Figure S2: Distribution of SAFHS participants across families** The 771 SAFHS participants included in this study came from 40 families. The distribution of number of subjects per family is shown in this bar chart here. Most pedigrees were complex.

**Supplementary Figure S3: Selection of participants from the AusDiab cohort** From the original study size of 11,247 Australian population we included a total of 653 non-diabetic subjects on whom complete phenotypic data including plasma lipidomic profiling was available. Of these, full detail on follow-up information was not available for 9 individuals. The remaining 644 individuals (233 of whom developed T2D during follow-up) were included in the validation analyses.


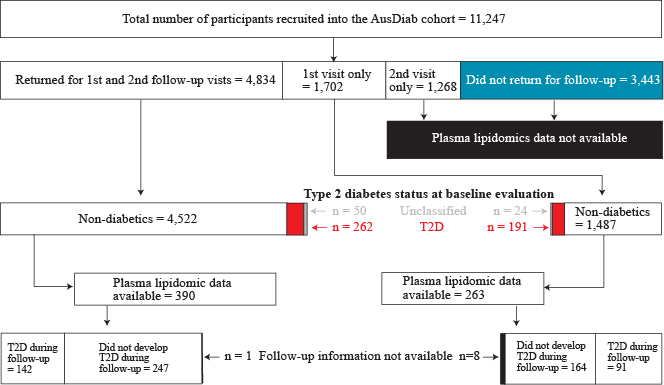


.

**Supplementary Figure S4.** **Development and accuracy of LRS in the SAFHS cohort** (A) Significant lipid species (FDR-corrected p<0·2) considered singly or in a stepwise regression. Diamonds and error bars show point and 95% confidence intervals, respectively. Species retained in the final model are color coded for consistency in the rest of the figure. All models are adjusted for age, age^2^, sex, age x sex interaction, age^2^ x sex interaction, systolic and diastolic blood pressures, waist circumference, body mass index, total serum cholesterol, serum high-density lipoprotein cholesterol, serum triglycerides and use of anti-lipid and anti-hypertensive drugs. (B) Retained lipid species and the LRS as predictors of T2D onset. (C) ROC curve for each component species and the LRS as predictors of incident T2D. AUC, area under the curve; SE, standard error; χ^2^, chi square; Ref, reference; P, statistical significance. (D) Observed probability of T2D based on time of follow-up and tertiles of LRS. P, statistical significance

** Supplementary Figure S5. Association of prediabetes with incident diabetes in the SAFHS cohort** Plot shows Kaplan-Meier survival curves and results from mixed effects Cox proportional model. NGT, normal glucose tolerance; IFG, impaired fasting glucose; IGT, impaired glucose tolerance.

**Supplementary Figure S6: Structure of the Decision Tree used to investigate the cost-effectiveness of screening strategies for T2D** The probability values are shown for the SAFHS data. Similar tree with different probability values was used for the AusDiab cohort. Decision node is shown as blue square, chance nodes are shown as green circles and terminal nodes are shown as red triangles. The probabilities used are described in details in Table S6. Node identifiers are shown in red font, probabilities in green font and payoffs in purple font. All payoffs are shown as Cost\Effectiveness at the end of 5 years. Costs are shown in 2015 US$ whereas effectiveness is measured in quality-adjusted life years (QALYs). Details of cost and effectiveness measures are provided in Table S7. A probability of # indicates a complementary probability value. Decision trees were constructed and analysed using TreeAge Pro (2015) software package.

**
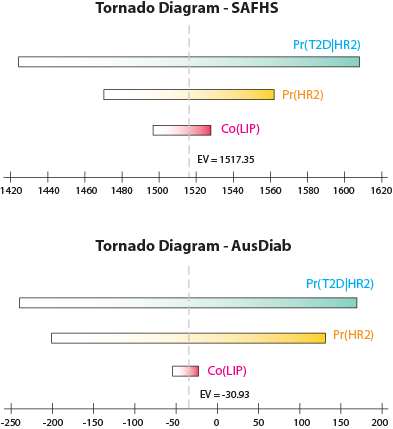
 Supplementary Figure S7. Sensitivity analyses for cost-effectiveness of T2D screening based on tornado diagrams.** Figure shows three most sensitive variables that influenced the cost-effectiveness of T2D screening using LRS and metformin supplementation. Top panel shows results for the SAFHS cohort while the lower panel shows results for the AusDiab cohort. The tornado analyses are based on net monetary benefits and assume a willingness-to-pay US$ 4450.12. Probability variables and their ranges used for sensitivity analyses are as described in Table S6. The range used for cost of lipidomic assays was 79$ - 109$. EV, expected value; Co(LIP), cost of lipidomic assays; Pr(), probability; |, conditional probability; T2D, incident type 2 diabetes; HR2, high risk group based on strategy #2 or #3

**Supplementary Table S1: Family relationships among pairs of SAFHS participants***

| **Relationship** | **Pairs** |
| --- | --- |
| Self | 768 |
| Identical sib pair | 1 |
| Parent-offspring | 422 |
| Siblings | 573 |
| Grandparent-grandchild | 104 |
| Avuncular | 1068 |
| Half siblings | 97 |
| 3rd degree | 1778 |
| 4th degree | 1604 |
| 5th degree | 923 |
| 6th degree | 295 |

*, Table shows the number of paired relationships out of a total of 296,835 paired relations among 771 participants

**Supplementary Table S2. Baseline characteristics of cohorts***

| Characteristic | **SAFHS cohort**  **(n = 771)†** | **AusDiab cohort**  **(n = 644)** |
| --- | --- | --- |
| Age at enrolment (y) | 35.6 (0.5) | 55.9 (0.5) |
| Average length of follow-up (y) | 11.9 (0.1) | 9.1 (0.1) |
| Females | 477 (61.9) | 314 (48.1) |
| Waist (cm) | 93.1 (0.6) | 93.3 (0.6) |
| Family history of diabetes | 200 (18.4) | 133 (20.4) |
| BMI (Kg/m^2^) | 29.0 (0.2) | 27.7 (0.2) |
| Systolic blood pressure (mmHg) | 117.1 (0.6) | 133.5 (0.7) |
| Diastolic blood pressure (mmHg) | 70.2 (0.4) | 72.8 (0.5) |
| Fasting glucose (mmol/l) | 4.9 (0.02) | 5.6 (0.02) |
| 2-hour post challenge glucose (mmol/l) | 5.7 (0.06) | 6.6 (0.07) |
| Prediabetes at baseline | 145 (18.8) | 260 (39.3) |
| Impaired fasting glucose only | 51 (6.6) | 73 (11.3) |
| Impaired glucose tolerance only | 63 (8.2) | 180 (28.0) |
| Impaired fasting glucose and impaired glucose tolerance | 31 (4.0) | 0 (0.0) |
| Total serum cholesterol (mg/dl) | 189.1 (1.3) | 224.3 (1.5) |
| Serum triglycerides (mg/dl) | 134.4 (3.3) | 153.5 (4.3) |
| HDL cholesterol (mg/dl) | 50.8 (0.5) | 54.4 (0.6) |
| Participants receiving lipid lowering medication | 13 (2.0) | 80 (12.3) |
| Participants receiving anti-hypertensive medications | 42 (5.5) | 136 (21.0) |

*, numbers indicate mean(SE) for continuous variables and n (%) for categorical variables

†, all proportions and means in the SAFHS cohort are adjusted for kinship structure

**Supplementary Table S3: Descriptive statistics of the plasma lipidome in SAFHS participants.** All values are in pmol/ml

| **No** | **Lipid Species** | **Median** | **IQR** |
| --- | --- | --- | --- |
| 1 | Cer(d18:0/16:0) | 48.5 | 21.9 |
| 2 | Cer(d18:0/18:0) | 69.2 | 43.1 |
| 3 | Cer(d18:0/20:0) | 30.1 | 16.9 |
| 4 | Cer(d18:0/22:0) | 133.1 | 72 |
| 5 | Cer(d18:0/24:0) | 183.8 | 105.7 |
| 6 | Cer(d18:0/24:1) | 109.7 | 67.1 |
| 7 | Cer(d18:1/16:0) | 349.4 | 108.3 |
| 8 | Cer(d18:1/18:0) | 151.9 | 74.4 |
| 9 | Cer(d18:1/20:0) | 132.7 | 57.6 |
| 10 | Cer(d18:1/22:0) | 923.1 | 340.9 |
| 11 | Cer(d18:1/24:0) | 2602.8 | 958.8 |
| 12 | Cer(d18:1/24:1) | 975.5 | 423.9 |
| 13 | HexCer(d18:1/16:0) | 902 | 313.1 |
| 14 | HexCer(d18:1/18:0) | 208.6 | 81 |
| 15 | HexCer(d18:1/20:0) | 231.3 | 100.6 |
| 16 | HexCer(d18:1/22:0) | 2441.1 | 971.8 |
| 17 | HexCer(d18:1/24:0) | 3052.5 | 1324 |
| 18 | HexCer(d18:1/24:1) | 2183.2 | 1029.6 |
| 19 | Hex2Cer(d18:1/16:0) | 4094.4 | 1351.2 |
| 20 | Hex2Cer(d18:1/18:0) | 86 | 33.9 |
| 21 | Hex2Cer(d18:1/20:0) | 71.9 | 37 |
| 22 | Hex2Cer(d18:1/22:0) | 344.9 | 141.9 |
| 23 | Hex2Cer(d18:1/24:1) | 966.3 | 374.5 |
| 24 | Hex2Cer(d18:1/24:0) | 281.2 | 110.5 |
| 25 | Hex3Cer(d18:1/16:0) | 794 | 227.7 |
| 26 | Hex3Cer(d18:1/18:0) | 104.5 | 37.8 |
| 27 | Hex3Cer(d18:1/20:0) | 32.5 | 15.2 |
| 28 | Hex3Cer(d18:1/22:0) | 147.1 | 63.2 |
| 29 | Hex3Cer(d18:1/24:1) | 245.8 | 89 |
| 30 | Hex3Cer(d18:1/24:0) | 140.6 | 52.9 |
| 31 | GM3(d18:1/16:0) | 725 | 203.5 |
| 32 | GM3(d18:1/18:0) | 326.9 | 138.2 |
| 33 | GM3(d18:1/20:0) | 183.2 | 67.2 |
| 34 | GM3(d18:1/22:0) | 508.3 | 195 |
| 35 | GM3(d18:1/24:1) | 571.4 | 234.2 |
| 36 | GM3(d18:1/24:0) | 387.7 | 183.5 |
| 37 | SM(31:1) | 271 | 109.9 |
| 38 | SM(32:1) | 10363.2 | 3531.2 |
| 39 | SM(32:2) | 1133.1 | 491.3 |
| 40 | SM(33:1) | 7526.2 | 2265.5 |
| 41 | SM(34:1) | 128517.6 | 27849 |
| 42 | SM(34:2) | 22864.8 | 6792.2 |
| 43 | SM(34:3) | 285.8 | 116.9 |
| 44 | SM(35:1) | 6187.5 | 1969.6 |
| 45 | SM(35:2) | 1415 | 475 |
| 46 | SM(36:1) | 31897 | 9651.9 |
| 47 | SM(36:2) | 20897.8 | 7355.8 |
| 48 | SM(36:3) | 3920.9 | 1686 |
| 49 | SM(38:1) | 17734 | 6192.9 |
| 50 | SM(38:2) | 7873.3 | 2802.1 |
| 51 | SM(39:1) | 10038.7 | 4717 |
| 52 | SM(41:1) | 25614.9 | 8533.3 |
| 53 | SM(41:2) | 20520.7 | 6037.4 |
| 54 | SM(42:1) | 31638.4 | 11011.9 |
| 55 | LPC(14:0) | 1010 | 511.1 |
| 56 | LPC(15:0) | 599.7 | 254.1 |
| 57 | LPC(16:0) | 80187.4 | 42137.8 |
| 58 | LPC(16:1) | 2785.6 | 981.6 |
| 59 | LPC(17:0) | 1638.6 | 1021.7 |
| 60 | LPC(17:1) | 317.7 | 122.3 |
| 61 | LPC(18:0) | 20035.3 | 7705.1 |
| 62 | LPC(18:1) | 28631.2 | 15754.1 |
| 63 | LPC(18:2) | 27945.1 | 11547.3 |
| 64 | LPC(18:3) | 452.5 | 254.4 |
| 65 | LPC(20:0) | 87.6 | 37.4 |
| 66 | LPC(20:1) | 213.2 | 82.2 |
| 67 | LPC(20:2) | 220.5 | 117.3 |
| 68 | LPC(20:3) | 2643.1 | 1075 |
| 69 | LPC(20:4) | 6689 | 2833.9 |
| 70 | LPC(20:5) | 335.3 | 220.6 |
| 71 | LPC(22:0) | 25.2 | 10.3 |
| 72 | LPC(22:1) | 11.7 | 5.5 |
| 73 | LPC(22:6) | 786.1 | 414.4 |
| 74 | LPC(24:0) | 45.8 | 18.2 |
| 75 | LPC(26:0) | 11.4 | 5.3 |
| 76 | PC(28:0) | 116.8 | 120.4 |
| 77 | PC(30:0) | 2102.2 | 1419.9 |
| 78 | PC(32:0) | 8085.3 | 2002.8 |
| 79 | PC(32:1) | 20902.5 | 10025 |
| 80 | PC(32:2) | 8864.2 | 3087.3 |
| 81 | PC(32:3) | 750.6 | 350.9 |
| 82 | PC(34:0) | 3664.1 | 1153.4 |
| 83 | PC(34:1) | 118368.9 | 27221.5 |
| 84 | PC(34:2) | 238061.9 | 47027.4 |
| 85 | PC(34:3) | 20229.8 | 7291.7 |
| 86 | PC(34:4) | 1867.4 | 1026.8 |
| 87 | PC(34:5) | 45 | 42.1 |
| 88 | PC(36:1) | 52183.9 | 14980 |
| 89 | PC(36:2) | 197959.7 | 39023.8 |
| 90 | PC(36:3) | 121051.3 | 21884.2 |
| 91 | PC(18:1_18:3) | 248.1 | 112.5 |
| 92 | PC(16:0_20:4) | 83246.1 | 28003.8 |
| 93 | PC(36:5) | 11221.5 | 6761.2 |
| 94 | PC(36:6) | 550.4 | 323.9 |
| 95 | PC(38:2) | 11340.4 | 4229.5 |
| 96 | PC(38:3) | 46184.7 | 19999.7 |
| 97 | PC(38:4) | 73611.6 | 27780.1 |
| 98 | PC(38:5) | 46048.4 | 13107 |
| 99 | PC(18:2_20:4) | 479 | 193.6 |
| 100 | PC(16:0_22:6) | 23208.5 | 8322.8 |
| 101 | PC(38:7) | 1468.8 | 643 |
| 102 | PC(40:5) | 13016.3 | 5031.7 |
| 103 | PC(40:6) | 11521.1 | 4968.9 |
| 104 | PC(40:7) | 4121.6 | 1759.6 |
| 105 | PC(29:0) | 11.9 | 5.1 |
| 106 | PC(31:0) | 543.4 | 277.4 |
| 107 | PC(31:1) | 1503.8 | 458.5 |
| 108 | PC(33:0) | 1076.1 | 274 |
| 109 | PC(33:1) | 2881.3 | 1034 |
| 110 | PC(33:2) | 3658.9 | 1328.1 |
| 111 | PC(33:3) | 81.1 | 43.3 |
| 112 | PC(35:0) | 251.6 | 156.7 |
| 113 | PC(35:1) | 457.6 | 142.5 |
| 114 | PC(35:2) | 698.7 | 216.6 |
| 115 | PC(35:3) | 1678.6 | 591.9 |
| 116 | PC(35:4) | 72.3 | 37.6 |
| 117 | PC(35:5) | 3 | 2.7 |
| 118 | PC(37:4) | 350.8 | 123.5 |
| 119 | PC(37:5) | 28.7 | 17.5 |
| 120 | PC(37:6) | 348.2 | 193.4 |
| 121 | PC(39:6) | 926.1 | 439 |
| 122 | LPC(O-20:0) | 22.2 | 9.5 |
| 123 | LPC(O-22:0) | 30.9 | 10.9 |
| 124 | LPC(O-22:1) | 23.9 | 12 |
| 125 | LPC(O-24:0) | 54.1 | 19.8 |
| 126 | LPC(O-24:1) | 53.3 | 29.6 |
| 127 | LPC(O-24:2) | 16.5 | 8.5 |
| 128 | PC(O-30:0) | 101.7 | 36.2 |
| 129 | PC(O-32:0) | 1379 | 355.6 |
| 130 | PC(O-32:1) | 276.1 | 106.9 |
| 131 | PC(P-32:0) | 894.9 | 265.4 |
| 132 | PC(O-32:2) | 50.4 | 27.7 |
| 133 | PC(P-32:1) | 149.7 | 58.7 |
| 134 | PC(O-34:0) | 507.9 | 153.4 |
| 135 | PC(O-34:1) | 3493.2 | 898.8 |
| 136 | PC(O-34:2) | 3495.8 | 1385 |
| 137 | PC(P-34:1) | 1644.4 | 498 |
| 138 | PC(P-34:2) | 4621.7 | 1639.9 |
| 139 | PC(O-34:4) | 251.8 | 126.2 |
| 140 | PC(O-35:4) | 147 | 84.1 |
| 141 | PC(O-36:0) | 67.9 | 28.5 |
| 142 | PC(O-36:1) | 707 | 235 |
| 143 | PC(O-36:2) | 230.4 | 80.4 |
| 144 | PC(O-36:3) | 4585.5 | 1391.9 |
| 145 | PC(P-36:2) | 2711.5 | 1195.2 |
| 146 | PC(O-36:4) | 10372.9 | 3442.8 |
| 147 | PC(O-36:5) | 492.5 | 202.7 |
| 148 | PC(P-36:5) | 349.3 | 212.6 |
| 149 | PC(O-38:4) | 9827.6 | 2624 |
| 150 | PC(O-38:5) | 10094.5 | 3058.8 |
| 151 | PC(P-38:5) | 4643.6 | 1331.1 |
| 152 | PC(P-40:5)/PC(O-40:6) | 2211 | 764.4 |
| 153 | SM(37:2) | 494.6 | 201.4 |
| 154 | PC(O-40:7) | 1399.3 | 465 |
| 155 | LPE(16:0) | 644.9 | 269.2 |
| 156 | LPE(18:0) | 958.1 | 403.6 |
| 157 | LPE(18:1) | 956.7 | 468.9 |
| 158 | LPE(18:2) | 705.1 | 306.6 |
| 159 | LPE(20:4) | 874.7 | 412.8 |
| 160 | LPE(22:6) | 429.1 | 180.9 |
| 161 | PE(32:1) | 121.1 | 125.6 |
| 162 | PE(34:1) | 1910.7 | 1284.7 |
| 163 | PE(34:2) | 3737.5 | 2646.9 |
| 164 | PE(34:3) | 229.2 | 174.5 |
| 165 | PE(36:1) | 1918.7 | 1376.8 |
| 166 | PE(36:2) | 10239.2 | 6669.1 |
| 167 | PE(36:3) | 3794.3 | 2356.1 |
| 168 | PE(36:4) | 4048.4 | 2497.5 |
| 169 | PE(36:5) | 175.5 | 137.3 |
| 170 | PE(38:3) | 2585.1 | 1671.2 |
| 171 | PE(38:4) | 11501 | 6346.8 |
| 172 | PE(38:5) | 4626.6 | 2248.6 |
| 173 | PE(38:6) | 3356.7 | 2306.8 |
| 174 | PE(40:5) | 929.2 | 637.2 |
| 175 | PE(40:6) | 1956.8 | 1497.4 |
| 176 | PE(40:7) | 480.6 | 312.1 |
| 177 | PE(35:1) | 177.1 | 107.2 |
| 178 | PE(35:2) | 237.5 | 144.7 |
| 179 | PE(O-34:1) | 241.6 | 100.7 |
| 180 | PE(O-34:2) | 181.1 | 99.7 |
| 181 | PE(O-36:2) | 353.8 | 150.1 |
| 182 | PE(O-36:3) | 324.1 | 165.6 |
| 183 | PE(O-36:4) | 1137.1 | 548 |
| 184 | PE(O-36:5) | 59.1 | 41.8 |
| 185 | PE(O-38:4) | 1448.9 | 730.5 |
| 186 | PE(O-38:5) | 1351.4 | 657.2 |
| 187 | PE(O-38:6) | 1757.5 | 670.9 |
| 188 | PE(O-40:5) | 384.6 | 163.4 |
| 189 | PE(O-40:6) | 738.9 | 293.3 |
| 190 | PE(O-40:7) | 361.2 | 140.6 |
| 191 | PE(P-34:1) | 125.8 | 58.6 |
| 192 | PE(P-34:2) | 239.5 | 131.9 |
| 193 | PE(P-36:4) | 871 | 472.5 |
| 194 | PE(P-38:6) | 351.7 | 146 |
| 195 | PE(P-36:1) | 145.7 | 74.1 |
| 196 | PE(P-36:2) | 647.3 | 288.6 |
| 197 | PE(P-38:4) | 1847.4 | 903.9 |
| 198 | PE(P-40:5) | 753.5 | 296.7 |
| 199 | PE(P-40:6) | 373.4 | 156.2 |
| 200 | PG(34:1) | 72.3 | 50.5 |
| 201 | PG(34:2) | 7.6 | 12.3 |
| 202 | PG(36:1) | 64.6 | 49.4 |
| 203 | PG(36:2) | 72.6 | 50.7 |
| 204 | PS(36:1) | 1331.4 | 1230.9 |
| 205 | PS(36:2) | 359.5 | 331 |
| 206 | PS(38:3) | 170.9 | 161 |
| 207 | PS(38:4) | 1359.8 | 1219.8 |
| 208 | PS(38:5) | 241.6 | 212.5 |
| 209 | PS(40:5) | 75.7 | 86 |
| 210 | PS(40:6) | 91.6 | 72.9 |
| 211 | PI(32:1) | 1006.8 | 1155.7 |
| 212 | PI(32:0) | 335.8 | 357.5 |
| 213 | PI(34:1) | 6909.3 | 4416.5 |
| 214 | PI(34:0) | 136 | 101.7 |
| 215 | PI(36:4) | 5736.6 | 3516.1 |
| 216 | PI(36:3) | 7366.2 | 3516.8 |
| 217 | PI(36:2) | 23036.1 | 9217.3 |
| 218 | PI(36:1) | 6269.7 | 3223.1 |
| 219 | PI(36:0) | 22.4 | 19.2 |
| 220 | PI(38:6) | 630.2 | 324.7 |
| 221 | PI(38:5) | 6030.2 | 2990.4 |
| 222 | PI(38:4) | 40011.9 | 16979.7 |
| 223 | PI(38:3) | 7501.5 | 4055 |
| 224 | PI(38:2) | 530.3 | 279 |
| 225 | PI(40:6) | 1149.2 | 597.7 |
| 226 | PI(40:5) | 1116.9 | 550.4 |
| 227 | PI(40:4) | 689.7 | 349.4 |
| 228 | Cholesterol | 874204.9 | 332303.5 |
| 229 | CE(14:0) | 15165.1 | 10754.4 |
| 230 | CE(15:0) | 14554.4 | 7640.1 |
| 231 | CE(16:0) | 411355.2 | 125134.7 |
| 232 | CE(16:1) | 112685.4 | 91034 |
| 233 | CE(16:2) | 4264.5 | 3083.2 |
| 234 | CE(17:0) | 9300 | 4116.8 |
| 235 | CE(17:1) | 14195.4 | 4774.3 |
| 236 | CE(18:0) | 24093.8 | 10851.6 |
| 237 | CE(18:1) | 334757.1 | 106924.7 |
| 238 | CE(18:2) | 745732.8 | 251916 |
| 239 | CE(18:3) | 71145.9 | 52168.5 |
| 240 | CE(20:1) | 287.5 | 115.8 |
| 241 | CE(20:2) | 509.3 | 188.1 |
| 242 | CE(20:3) | 15976.9 | 8944.9 |
| 243 | CE(20:4) | 419321 | 228637.8 |
| 244 | CE(20:5) | 34895 | 30837.2 |
| 245 | CE(22:0) | 151.3 | 111 |
| 246 | CE(22:1) | 109.6 | 54.3 |
| 247 | CE(22:4) | 431 | 214 |
| 248 | CE(22:5) | 5353.3 | 3166.8 |
| 249 | CE(22:6) | 37381.3 | 24557.9 |
| 250 | CE(24:0) | 121.1 | 87.5 |
| 251 | CE(24:1) | 147.5 | 84.6 |
| 252 | CE(24:4) | 20.8 | 12.4 |
| 253 | CE(24:5) | 41.7 | 22.7 |
| 254 | CE(24:6) | 47.5 | 36.6 |
| 255 | DG(14:0_16:0) | 140.8 | 178.5 |
| 256 | DG(14:0_18:1) | 482.9 | 580.7 |
| 257 | DG(14:0_18:2) | 267.3 | 294.1 |
| 258 | DG(16:0_16:0) | 583.7 | 667 |
| 259 | DG(16:0_18:0) | 509.1 | 480.6 |
| 260 | DG(16:0_18:1) | 4807.9 | 4170.2 |
| 261 | DG(16:0_18:2) | 2886 | 2497.4 |
| 262 | DG(16:0_20:3) | 124.9 | 120.4 |
| 263 | DG(16:0_20:4) | 271.3 | 291.2 |
| 264 | DG(16:0_22:5) | 91 | 84 |
| 265 | DG(16:0_22:6) | 63.8 | 77.2 |
| 266 | DG(16:1_18:1) | 2286.5 | 2048.9 |
| 267 | DG(16:1_18:0) | 141.4 | 159.3 |
| 268 | DG(18:0_18:1) | 1410.2 | 1175.9 |
| 269 | DG(18:0_18:2) | 749.9 | 603.3 |
| 270 | DG(18:0_20:4) | 147.1 | 98.5 |
| 271 | DG(18:1_18:1) | 7370.7 | 5705.6 |
| 272 | DG(18:1_18:2) | 9323.7 | 7354 |
| 273 | DG(18:1_18:3) | 1006 | 780 |
| 274 | DG(18:1_20:3) | 414.1 | 303.2 |
| 275 | DG(18:1_20:4) | 1138.1 | 950.6 |
| 276 | DG(18:2_18:2) | 1838.1 | 1549.2 |
| 277 | TG(14:0_16:0_18:2) | 6408.3 | 8126.7 |
| 278 | TG(14:0_16:1_18:1) | 7270.8 | 7491.5 |
| 279 | TG(14:0_16:1_18:2) | 1937.3 | 1915.4 |
| 280 | TG(14:0_18:0_18:1) | 578.2 | 688.6 |
| 281 | TG(14:0_18:2_18:2) | 1159.4 | 1140.9 |
| 282 | TG(14:1_16:0_18:1) | 1724.5 | 2051.6 |
| 283 | TG(14:1_16:1_18:0) | 6207.1 | 7469.2 |
| 284 | TG(14:1_18:0_18:2) | 486.1 | 392.7 |
| 285 | TG(14:1_18:1_18:1) | 5336.4 | 4196.8 |
| 286 | TG(15:0_16:0_18:1) | 1050.2 | 1167.1 |
| 287 | TG(15:0_18:1_18:1) | 978.2 | 751.2 |
| 288 | TG(16:0_16:0_16:0) | 2430.8 | 3901 |
| 289 | TG(16:0_16:0_18:0) | 2773 | 3934.4 |
| 290 | TG(16:0_16:0_18:1) | 12539.5 | 12889 |
| 291 | TG(16:0_16:0_18:2) | 11621.4 | 11489.2 |
| 292 | TG(16:0_16:1_18:1) | 41750.3 | 36973 |
| 293 | TG(16:0_18:0_18:1) | 11692.5 | 11172 |
| 294 | TG(16:0_18:1_18:1) | 114641 | 79060.2 |
| 295 | TG(16:0_18:1_18:2) | 77518.6 | 51410.2 |
| 296 | TG(16:0_18:2_18:2) | 19047.2 | 15298.1 |
| 297 | TG(16:1_16:1_16:1) | 669.1 | 736.3 |
| 298 | TG(16:1_16:1_18:0) | 698.2 | 734.5 |
| 299 | TG(16:1_16:1_18:1) | 6970.7 | 5830.1 |
| 300 | TG(16:1_18:1_18:1) | 11088.4 | 7964 |
| 301 | TG(16:1_18:1_18:2) | 18302.1 | 13249.2 |
| 302 | TG(16:0_16:1_17:0) | 2649 | 3134.5 |
| 303 | TG(16:0_17:0_18:0) | 122.2 | 184.8 |
| 304 | TG(14:0_17:0_18:1) | 1941.6 | 2085.2 |
| 305 | TG(16:0_17:0_18:1) | 1848.7 | 1730 |
| 306 | TG(16:1_17:0_18:1) | 6061.6 | 4939.7 |
| 307 | TG(17:0_18:1_18:1) | 3066.6 | 2350.5 |
| 308 | TG(16:0_17:0_18:2) | 4228.7 | 3764.5 |
| 309 | TG(18:0_18:0_18:1) | 861.2 | 1015.7 |
| 310 | TG(18:0_18:1_18:1) | 9690.5 | 7619.9 |
| 311 | TG(18:0_18:2_18:2) | 1585.9 | 1220.2 |
| 312 | TG(14:0_16:0_18:1) | 8645.1 | 10629.3 |
| 313 | TG(18:1_18:1_18:1) | 21637.1 | 13373.6 |
| 314 | TG(18:1_18:1_18:2) | 15726.9 | 11163.5 |
| 315 | TG(18:1_18:1_20:4) | 2351.6 | 1412.9 |
| 316 | TG(18:1_18:1_22:6) | 1454.7 | 912.2 |
| 317 | TG(18:1_18:2_18:2) | 10081.9 | 7189.8 |
| 318 | TG(18:2_18:2_18:2) | 1063 | 962.5 |
| 319 | TG(18:2_18:2_20:4) | 493.1 | 414.6 |

IQR, inter-quartile range

**Supplementary Table S4: Results of mixed effects Cox proportional hazards for association of each lipid species with onset of T2D in the SAFHS cohort.**

| **No** | **Lipid Species** | **β** | **RH** | **SE(β)** | **Z** | **Nom-P** | **FDR-P** | **REV** |
| --- | --- | --- | --- | --- | --- | --- | --- | --- |
| 1 | Cer(d18:0/16:0) | 0.2925 | 1.34 | 0.1108 | 2.64 | 0.0083 | 0.9992 | 0.2123 |
| 2 | Cer(d18:0/18:0) | 0.5150 | 1.67 | 0.1201 | 4.29 | 0.0000 | 0.0056 | 0.1899 |
| 3 | Cer(d18:0/20:0) | 0.3135 | 1.37 | 0.1187 | 2.64 | 0.0082 | 0.9992 | 0.3819 |
| 4 | Cer(d18:0/22:0) | 0.4590 | 1.58 | 0.1190 | 3.86 | 0.0001 | 0.0366 | 0.3287 |
| 5 | Cer(d18:0/24:0) | 0.3796 | 1.46 | 0.1139 | 3.33 | 0.0009 | 0.2640 | 0.3676 |
| 6 | Cer(d18:0/24:1) | 0.3924 | 1.48 | 0.1169 | 3.36 | 0.0008 | 0.2437 | 0.4348 |
| 7 | Cer(d18:1/16:0) | 0.3071 | 1.36 | 0.1319 | 2.33 | 0.0199 | 0.9992 | 0.3717 |
| 8 | Cer(d18:1/18:0) | 0.4593 | 1.58 | 0.1322 | 3.47 | 0.0005 | 0.1587 | 0.4619 |
| 9 | Cer(d18:1/20:0) | 0.3578 | 1.43 | 0.1290 | 2.77 | 0.0055 | 0.9992 | 0.4564 |
| 10 | Cer(d18:1/22:0) | 0.4033 | 1.50 | 0.1365 | 2.95 | 0.0031 | 0.9461 | 0.3538 |
| 11 | Cer(d18:1/24:0) | 0.2047 | 1.23 | 0.1363 | 1.50 | 0.1332 | 0.9992 | 0.3891 |
| 12 | Cer(d18:1/24:1) | 0.2554 | 1.29 | 0.1269 | 2.01 | 0.0442 | 0.9992 | 0.4786 |
| 13 | HexCer(d18:1/16:0) | 0.2835 | 1.33 | 0.1121 | 2.53 | 0.0114 | 0.9992 | 0.3673 |
| 14 | HexCer(d18:1/18:0) | 0.0780 | 1.08 | 0.1093 | 0.71 | 0.4755 | 0.9992 | 0.3911 |
| 15 | HexCer(d18:1/20:0) | 0.1356 | 1.15 | 0.1132 | 1.20 | 0.2309 | 0.9992 | 0.4102 |
| 16 | HexCer(d18:1/22:0) | 0.1219 | 1.13 | 0.1121 | 1.09 | 0.2772 | 0.9992 | 0.3998 |
| 17 | HexCer(d18:1/24:0) | 0.0797 | 1.08 | 0.1159 | 0.69 | 0.4919 | 0.9992 | 0.4087 |
| 18 | HexCer(d18:1/24:1) | 0.0551 | 1.06 | 0.1112 | 0.50 | 0.6202 | 0.9992 | 0.4132 |
| 19 | Hex2Cer(d18:1/16:0) | -0.0140 | 0.99 | 0.1075 | -0.13 | 0.8963 | 0.9992 | 0.4104 |
| 20 | Hex2Cer(d18:1/18:0) | 0.0821 | 1.09 | 0.1093 | 0.75 | 0.4524 | 0.9992 | 0.3780 |
| 21 | Hex2Cer(d18:1/20:0) | 0.0529 | 1.05 | 0.1058 | 0.50 | 0.6169 | 0.9992 | 0.3984 |
| 22 | Hex2Cer(d18:1/22:0) | 0.2496 | 1.28 | 0.1086 | 2.30 | 0.0215 | 0.9992 | 0.2823 |
| 23 | Hex2Cer(d18:1/24:1) | 0.0392 | 1.04 | 0.1115 | 0.35 | 0.7248 | 0.9992 | 0.4017 |
| 24 | Hex2Cer(d18:1/24:0) | 0.2716 | 1.31 | 0.1128 | 2.41 | 0.0161 | 0.9992 | 0.2877 |
| 25 | Hex3Cer(d18:1/16:0) | -0.1082 | 0.90 | 0.1187 | -0.91 | 0.3617 | 0.9992 | 0.4224 |
| 26 | Hex3Cer(d18:1/18:0) | -0.0637 | 0.94 | 0.1164 | -0.55 | 0.5843 | 0.9992 | 0.4321 |
| 27 | Hex3Cer(d18:1/20:0) | -0.1192 | 0.89 | 0.1052 | -1.13 | 0.2569 | 0.9992 | 0.4534 |
| 28 | Hex3Cer(d18:1/22:0) | -0.1010 | 0.90 | 0.1138 | -0.89 | 0.3747 | 0.9992 | 0.4229 |
| 29 | Hex3Cer(d18:1/24:1) | -0.1211 | 0.89 | 0.1172 | -1.03 | 0.3017 | 0.9992 | 0.4054 |
| 30 | Hex3Cer(d18:1/24:0) | -0.1123 | 0.89 | 0.1215 | -0.92 | 0.3553 | 0.9992 | 0.4282 |
| 31 | GM3(d18:1/16:0) | 0.1331 | 1.14 | 0.1166 | 1.14 | 0.2535 | 0.9992 | 0.4122 |
| 32 | GM3(d18:1/18:0) | 0.1498 | 1.16 | 0.1220 | 1.23 | 0.2197 | 0.9992 | 0.4067 |
| 33 | GM3(d18:1/20:0) | 0.1658 | 1.18 | 0.1145 | 1.45 | 0.1473 | 0.9992 | 0.3838 |
| 34 | GM3(d18:1/22:0) | 0.1976 | 1.22 | 0.1152 | 1.72 | 0.0862 | 0.9992 | 0.3266 |
| 35 | GM3(d18:1/24:1) | -0.0012 | 1.00 | 0.1192 | -0.01 | 0.9922 | 0.9992 | 0.4095 |
| 36 | GM3(d18:1/24:0) | -0.0518 | 0.95 | 0.1231 | -0.42 | 0.6739 | 0.9992 | 0.4275 |
| 37 | SM(31:1) | -0.0148 | 0.99 | 0.1224 | -0.12 | 0.9040 | 0.9992 | 0.4095 |
| 38 | SM(32:1) | 0.2224 | 1.25 | 0.1268 | 1.75 | 0.0793 | 0.9992 | 0.4138 |
| 39 | SM(32:2) | -0.2822 | 0.75 | 0.1555 | -1.81 | 0.0696 | 0.9992 | 0.4625 |
| 40 | SM(33:1) | 0.1787 | 1.20 | 0.1220 | 1.47 | 0.1429 | 0.9992 | 0.4188 |
| 41 | SM(34:1) | 0.0887 | 1.09 | 0.1317 | 0.67 | 0.5007 | 0.9992 | 0.4097 |
| 42 | SM(34:2) | -0.1012 | 0.90 | 0.1482 | -0.68 | 0.4949 | 0.9992 | 0.4185 |
| 43 | SM(34:3) | -0.1975 | 0.82 | 0.1319 | -1.50 | 0.1342 | 0.9992 | 0.4414 |
| 44 | SM(35:1) | 0.0899 | 1.09 | 0.1196 | 0.75 | 0.4523 | 0.9992 | 0.4981 |
| 45 | SM(35:2) | -0.0924 | 0.91 | 0.1201 | -0.77 | 0.4414 | 0.9992 | 0.4068 |
| 46 | SM(36:1) | 0.2505 | 1.28 | 0.1277 | 1.96 | 0.0498 | 0.9992 | 0.4417 |
| 47 | SM(36:2) | -0.0481 | 0.95 | 0.1306 | -0.37 | 0.7125 | 0.9992 | 0.4072 |
| 48 | SM(36:3) | -0.2156 | 0.81 | 0.1239 | -1.74 | 0.0819 | 0.9992 | 0.4108 |
| 49 | SM(38:1) | 0.2419 | 1.27 | 0.1121 | 2.16 | 0.0310 | 0.9992 | 0.4151 |
| 50 | SM(38:2) | -0.2300 | 0.79 | 0.1136 | -2.02 | 0.0429 | 0.9992 | 0.3712 |
| 51 | SM(39:1) | 0.0910 | 1.10 | 0.1091 | 0.83 | 0.4041 | 0.9992 | 0.3969 |
| 52 | SM(41:1) | 0.2643 | 1.30 | 0.1278 | 2.07 | 0.0387 | 0.9992 | 0.4044 |
| 53 | SM(41:2) | -0.0234 | 0.98 | 0.1221 | -0.19 | 0.8483 | 0.9992 | 0.4098 |
| 54 | SM(42:1) | 0.1922 | 1.21 | 0.1299 | 1.48 | 0.1389 | 0.9992 | 0.4142 |
| 55 | LPC(14:0) | 0.0478 | 1.05 | 0.1101 | 0.43 | 0.6638 | 0.9992 | 0.3926 |
| 56 | LPC(15:0) | -0.0767 | 0.93 | 0.1028 | -0.75 | 0.4557 | 0.9992 | 0.4221 |
| 57 | LPC(16:0) | -0.0346 | 0.97 | 0.1051 | -0.33 | 0.7418 | 0.9992 | 0.3946 |
| 58 | LPC(16:1) | -0.1276 | 0.88 | 0.1072 | -1.19 | 0.2340 | 0.9992 | 0.4009 |
| 59 | LPC(17:0) | -0.1486 | 0.86 | 0.1036 | -1.43 | 0.1515 | 0.9992 | 0.4332 |
| 60 | LPC(17:1) | -0.2168 | 0.81 | 0.1059 | -2.05 | 0.0407 | 0.9992 | 0.3776 |
| 61 | LPC(18:0) | -0.0798 | 0.92 | 0.1055 | -0.76 | 0.4497 | 0.9992 | 0.4117 |
| 62 | LPC(18:1) | -0.2302 | 0.79 | 0.1070 | -2.15 | 0.0315 | 0.9992 | 0.3941 |
| 63 | LPC(18:2) | -0.1367 | 0.87 | 0.1191 | -1.15 | 0.2513 | 0.9992 | 0.4094 |
| 64 | LPC(18:3) | -0.1158 | 0.89 | 0.1128 | -1.03 | 0.3048 | 0.9992 | 0.4335 |
| 65 | LPC(20:0) | -0.2374 | 0.79 | 0.1136 | -2.09 | 0.0366 | 0.9992 | 0.4254 |
| 66 | LPC(20:1) | -0.2732 | 0.76 | 0.1113 | -2.45 | 0.0141 | 0.9992 | 0.3514 |
| 67 | LPC(20:2) | -0.1635 | 0.85 | 0.1041 | -1.57 | 0.1164 | 0.9992 | 0.3846 |
| 68 | LPC(20:3) | 0.0850 | 1.09 | 0.1120 | 0.76 | 0.4475 | 0.9992 | 0.4216 |
| 69 | LPC(20:4) | -0.0712 | 0.93 | 0.1066 | -0.67 | 0.5040 | 0.9992 | 0.4001 |
| 70 | LPC(20:5) | -0.0600 | 0.94 | 0.1114 | -0.54 | 0.5900 | 0.9992 | 0.4089 |
| 71 | LPC(22:0) | -0.2084 | 0.81 | 0.1117 | -1.87 | 0.0621 | 0.9992 | 0.4363 |
| 72 | LPC(22:1) | -0.2344 | 0.79 | 0.1161 | -2.02 | 0.0434 | 0.9992 | 0.4182 |
| 73 | LPC(22:6) | -0.1372 | 0.87 | 0.1031 | -1.33 | 0.1831 | 0.9992 | 0.3987 |
| 74 | LPC(24:0) | -0.1447 | 0.87 | 0.1181 | -1.23 | 0.2203 | 0.9992 | 0.4059 |
| 75 | LPC(26:0) | -0.2349 | 0.79 | 0.1117 | -2.10 | 0.0354 | 0.9992 | 0.4200 |
| 76 | PC(28:0) | 0.0885 | 1.09 | 0.1122 | 0.79 | 0.4304 | 0.9992 | 0.3748 |
| 77 | PC(30:0) | 0.1511 | 1.16 | 0.1165 | 1.30 | 0.1948 | 0.9992 | 0.3679 |
| 78 | PC(32:0) | 0.1402 | 1.15 | 0.1056 | 1.33 | 0.1843 | 0.9992 | 0.3810 |
| 79 | PC(32:1) | 0.2191 | 1.24 | 0.1214 | 1.80 | 0.0712 | 0.9992 | 0.4184 |
| 80 | PC(32:2) | 0.0998 | 1.10 | 0.1404 | 0.71 | 0.4773 | 0.9992 | 0.3890 |
| 81 | PC(32:3) | -0.1727 | 0.84 | 0.1244 | -1.39 | 0.1652 | 0.9992 | 0.4535 |
| 82 | PC(34:0) | 0.0544 | 1.06 | 0.1106 | 0.49 | 0.6228 | 0.9992 | 0.3993 |
| 83 | PC(34:1) | 0.1147 | 1.12 | 0.1089 | 1.05 | 0.2920 | 0.9992 | 0.4027 |
| 84 | PC(34:2) | 0.1437 | 1.15 | 0.1014 | 1.42 | 0.1563 | 0.9992 | 0.3510 |
| 85 | PC(34:3) | -0.0253 | 0.98 | 0.1317 | -0.19 | 0.8476 | 0.9992 | 0.4139 |
| 86 | PC(34:4) | 0.0079 | 1.01 | 0.1300 | 0.06 | 0.9514 | 0.9992 | 0.4077 |
| 87 | PC(34:5) | -0.0057 | 0.99 | 0.1172 | -0.05 | 0.9613 | 0.9992 | 0.4107 |
| 88 | PC(36:1) | 0.1353 | 1.14 | 0.1174 | 1.15 | 0.2493 | 0.9992 | 0.4044 |
| 89 | PC(36:2) | 0.1345 | 1.14 | 0.1048 | 1.28 | 0.1993 | 0.9992 | 0.3506 |
| 90 | PC(36:3) | 0.1589 | 1.17 | 0.1118 | 1.42 | 0.1553 | 0.9992 | 0.3799 |
| 91 | PC(18:1_18:3) | 0.0420 | 1.04 | 0.1090 | 0.39 | 0.7002 | 0.9992 | 0.3887 |
| 92 | PC(16:0_20:4) | 0.0704 | 1.07 | 0.1065 | 0.66 | 0.5084 | 0.9992 | 0.3890 |
| 93 | PC(36:5) | 0.0476 | 1.05 | 0.1196 | 0.40 | 0.6905 | 0.9992 | 0.4037 |
| 94 | PC(36:6) | -0.0212 | 0.98 | 0.1268 | -0.17 | 0.8675 | 0.9992 | 0.4144 |
| 95 | PC(38:2) | 0.0492 | 1.05 | 0.1179 | 0.42 | 0.6764 | 0.9992 | 0.4082 |
| 96 | PC(38:3) | 0.2412 | 1.27 | 0.1205 | 2.00 | 0.0454 | 0.9992 | 0.3759 |
| 97 | PC(38:4) | -0.0715 | 0.93 | 0.1088 | -0.66 | 0.5107 | 0.9992 | 0.4138 |
| 98 | PC(38:5) | -0.1726 | 0.84 | 0.1063 | -1.62 | 0.1043 | 0.9992 | 0.4415 |
| 99 | PC(18:2_20:4) | 0.0895 | 1.09 | 0.1134 | 0.79 | 0.4302 | 0.9992 | 0.3864 |
| 100 | PC(16:0_22:6) | -0.0079 | 0.99 | 0.1061 | -0.07 | 0.9410 | 0.9992 | 0.4092 |
| 101 | PC(38:7) | -0.1186 | 0.89 | 0.1184 | -1.00 | 0.3165 | 0.9992 | 0.4341 |
| 102 | PC(40:5) | 0.0035 | 1.00 | 0.1153 | 0.03 | 0.9757 | 0.9992 | 0.4092 |
| 103 | PC(40:6) | 0.0148 | 1.01 | 0.1087 | 0.14 | 0.8915 | 0.9992 | 0.4103 |
| 104 | PC(40:7) | -0.2631 | 0.77 | 0.1061 | -2.48 | 0.0132 | 0.9992 | 0.3780 |
| 105 | PC(29:0) | 0.3052 | 1.36 | 0.1166 | 2.62 | 0.0089 | 0.9992 | 0.3794 |
| 106 | PC(31:0) | 0.2292 | 1.26 | 0.1056 | 2.17 | 0.0300 | 0.9992 | 0.3106 |
| 107 | PC(31:1) | 0.1771 | 1.19 | 0.1185 | 1.49 | 0.1349 | 0.9992 | 0.4126 |
| 108 | PC(33:0) | 0.1340 | 1.14 | 0.1063 | 1.26 | 0.2073 | 0.9992 | 0.3357 |
| 109 | PC(33:1) | 0.1073 | 1.11 | 0.1042 | 1.03 | 0.3030 | 0.9992 | 0.4019 |
| 110 | PC(33:2) | 0.1392 | 1.15 | 0.1125 | 1.24 | 0.2158 | 0.9992 | 0.3764 |
| 111 | PC(33:3) | -0.0209 | 0.98 | 0.1176 | -0.18 | 0.8586 | 0.9992 | 0.4155 |
| 112 | PC(35:0) | 0.0472 | 1.05 | 0.1013 | 0.47 | 0.6414 | 0.9992 | 0.3835 |
| 113 | PC(35:1) | -0.0138 | 0.99 | 0.1089 | -0.13 | 0.8988 | 0.9992 | 0.4097 |
| 114 | PC(35:2) | -0.0258 | 0.97 | 0.1048 | -0.25 | 0.8058 | 0.9992 | 0.4159 |
| 115 | PC(35:3) | 0.0354 | 1.04 | 0.1098 | 0.32 | 0.7469 | 0.9992 | 0.3976 |
| 116 | PC(35:4) | 0.1264 | 1.13 | 0.1065 | 1.19 | 0.2351 | 0.9992 | 0.3870 |
| 117 | PC(35:5) | 0.1085 | 1.11 | 0.1083 | 1.00 | 0.3162 | 0.9992 | 0.4080 |
| 118 | PC(37:4) | -0.0572 | 0.94 | 0.1016 | -0.56 | 0.5734 | 0.9992 | 0.4212 |
| 119 | PC(37:5) | -0.0658 | 0.94 | 0.1027 | -0.64 | 0.5216 | 0.9992 | 0.4065 |
| 120 | PC(37:6) | 0.0372 | 1.04 | 0.1091 | 0.34 | 0.7334 | 0.9992 | 0.4078 |
| 121 | PC(39:6) | -0.1076 | 0.90 | 0.1045 | -1.03 | 0.3032 | 0.9992 | 0.3993 |
| 122 | LPC(O-20:0) | -0.3761 | 0.69 | 0.1123 | -3.35 | 0.0008 | 0.2487 | 0.4724 |
| 123 | LPC(O-22:0) | -0.1310 | 0.88 | 0.1040 | -1.26 | 0.2078 | 0.9992 | 0.4671 |
| 124 | LPC(O-22:1) | -0.3992 | 0.67 | 0.1091 | -3.66 | 0.0003 | 0.0792 | 0.3455 |
| 125 | LPC(O-24:0) | -0.0876 | 0.92 | 0.1043 | -0.84 | 0.4013 | 0.9992 | 0.4414 |
| 126 | LPC(O-24:1) | -0.1439 | 0.87 | 0.1022 | -1.41 | 0.1590 | 0.9992 | 0.3949 |
| 127 | LPC(O-24:2) | -0.2843 | 0.75 | 0.1106 | -2.57 | 0.0102 | 0.9992 | 0.4590 |
| 128 | PC(O-30:0) | -0.0244 | 0.98 | 0.1097 | -0.22 | 0.8239 | 0.9992 | 0.4190 |
| 129 | PC(O-32:0) | 0.0513 | 1.05 | 0.1097 | 0.47 | 0.6401 | 0.9992 | 0.3902 |
| 130 | PC(O-32:1) | -0.1547 | 0.86 | 0.1155 | -1.34 | 0.1804 | 0.9992 | 0.4398 |
| 131 | PC(P-32:0) | -0.0626 | 0.94 | 0.1151 | -0.54 | 0.5864 | 0.9992 | 0.4276 |
| 132 | PC(O-32:2) | 0.0505 | 1.05 | 0.1027 | 0.49 | 0.6229 | 0.9992 | 0.3968 |
| 133 | PC(P-32:1) | -0.2525 | 0.78 | 0.1128 | -2.24 | 0.0251 | 0.9992 | 0.3731 |
| 134 | PC(O-34:0) | 0.1692 | 1.18 | 0.1074 | 1.58 | 0.1152 | 0.9992 | 0.3471 |
| 135 | PC(O-34:1) | -0.1226 | 0.88 | 0.1164 | -1.05 | 0.2921 | 0.9992 | 0.4194 |
| 136 | PC(O-34:2) | -0.0031 | 1.00 | 0.1051 | -0.03 | 0.9762 | 0.9992 | 0.4101 |
| 137 | PC(P-34:1) | -0.3345 | 0.72 | 0.1265 | -2.64 | 0.0082 | 0.9992 | 0.5073 |
| 138 | PC(P-34:2) | -0.1035 | 0.90 | 0.1119 | -0.92 | 0.3551 | 0.9992 | 0.4249 |
| 139 | PC(O-34:4) | -0.1194 | 0.89 | 0.1076 | -1.11 | 0.2672 | 0.9992 | 0.4287 |
| 140 | PC(O-35:4) | -0.0707 | 0.93 | 0.1075 | -0.66 | 0.5109 | 0.9992 | 0.4253 |
| 141 | PC(O-36:0) | 0.0727 | 1.08 | 0.1056 | 0.69 | 0.4914 | 0.9992 | 0.4068 |
| 142 | PC(O-36:1) | -0.1327 | 0.88 | 0.1142 | -1.16 | 0.2452 | 0.9992 | 0.3907 |
| 143 | PC(O-36:2) | -0.1489 | 0.86 | 0.1120 | -1.33 | 0.1835 | 0.9992 | 0.4384 |
| 144 | PC(O-36:3) | 0.0642 | 1.07 | 0.1049 | 0.61 | 0.5405 | 0.9992 | 0.3797 |
| 145 | PC(P-36:2) | -0.2075 | 0.81 | 0.1111 | -1.87 | 0.0619 | 0.9992 | 0.4378 |
| 146 | PC(O-36:4) | 0.0808 | 1.08 | 0.1045 | 0.77 | 0.4393 | 0.9992 | 0.3651 |
| 147 | PC(O-36:5) | 0.0423 | 1.04 | 0.1112 | 0.38 | 0.7038 | 0.9992 | 0.4014 |
| 148 | PC(P-36:5) | -0.0042 | 1.00 | 0.1106 | -0.04 | 0.9694 | 0.9992 | 0.4097 |
| 149 | PC(O-38:4) | -0.0131 | 0.99 | 0.1066 | -0.12 | 0.9021 | 0.9992 | 0.4140 |
| 150 | PC(O-38:5) | -0.0334 | 0.97 | 0.1058 | -0.32 | 0.7525 | 0.9992 | 0.4290 |
| 151 | PC(P-38:5) | -0.0422 | 0.96 | 0.1084 | -0.39 | 0.6969 | 0.9992 | 0.4205 |
| 152 | PC(P-40:5)/PC(O-40:6) | -0.0413 | 0.96 | 0.1052 | -0.39 | 0.6947 | 0.9992 | 0.4201 |
| 153 | SM(37:2) | -0.0417 | 0.96 | 0.1251 | -0.33 | 0.7389 | 0.9992 | 0.4103 |
| 154 | PC(O-40:7) | 0.0179 | 1.02 | 0.1002 | 0.18 | 0.8582 | 0.9992 | 0.4032 |
| 155 | LPE(16:0) | -0.0359 | 0.96 | 0.1099 | -0.33 | 0.7435 | 0.9992 | 0.4086 |
| 156 | LPE(18:0) | -0.0635 | 0.94 | 0.1153 | -0.55 | 0.5816 | 0.9992 | 0.4155 |
| 157 | LPE(18:1) | -0.0541 | 0.95 | 0.1106 | -0.49 | 0.6250 | 0.9992 | 0.4084 |
| 158 | LPE(18:2) | -0.0153 | 0.98 | 0.1090 | -0.14 | 0.8881 | 0.9992 | 0.4088 |
| 159 | LPE(20:4) | 0.0135 | 1.01 | 0.1033 | 0.13 | 0.8956 | 0.9992 | 0.4116 |
| 160 | LPE(22:6) | 0.0410 | 1.04 | 0.1017 | 0.40 | 0.6867 | 0.9992 | 0.4172 |
| 161 | PE(32:1) | 0.2509 | 1.29 | 0.1166 | 2.15 | 0.0314 | 0.9992 | 0.4057 |
| 162 | PE(34:1) | 0.3421 | 1.41 | 0.1234 | 2.77 | 0.0056 | 0.9992 | 0.4125 |
| 163 | PE(34:2) | 0.3267 | 1.39 | 0.1199 | 2.72 | 0.0064 | 0.9992 | 0.3815 |
| 164 | PE(34:3) | 0.1786 | 1.20 | 0.1180 | 1.51 | 0.1303 | 0.9992 | 0.4093 |
| 165 | PE(36:1) | 0.4950 | 1.64 | 0.1403 | 3.53 | 0.0004 | 0.1285 | 0.3491 |
| 166 | PE(36:2) | 0.3943 | 1.48 | 0.1322 | 2.98 | 0.0028 | 0.8593 | 0.3678 |
| 167 | PE(36:3) | 0.2804 | 1.32 | 0.1191 | 2.35 | 0.0186 | 0.9992 | 0.3352 |
| 168 | PE(36:4) | 0.3324 | 1.39 | 0.1229 | 2.71 | 0.0068 | 0.9992 | 0.3790 |
| 169 | PE(36:5) | 0.1451 | 1.16 | 0.1206 | 1.20 | 0.2292 | 0.9992 | 0.3895 |
| 170 | PE(38:3) | 0.4700 | 1.60 | 0.1394 | 3.37 | 0.0007 | 0.2306 | 0.4159 |
| 171 | PE(38:4) | 0.3635 | 1.44 | 0.1324 | 2.75 | 0.0060 | 0.9992 | 0.3958 |
| 172 | PE(38:5) | 0.2061 | 1.23 | 0.1193 | 1.73 | 0.0840 | 0.9992 | 0.3788 |
| 173 | PE(38:6) | 0.3417 | 1.41 | 0.1221 | 2.80 | 0.0051 | 0.9992 | 0.3889 |
| 174 | PE(40:5) | 0.3098 | 1.36 | 0.1298 | 2.39 | 0.0170 | 0.9992 | 0.4448 |
| 175 | PE(40:6) | 0.3910 | 1.48 | 0.1332 | 2.94 | 0.0033 | 0.9936 | 0.4135 |
| 176 | PE(40:7) | 0.2384 | 1.27 | 0.1178 | 2.02 | 0.0429 | 0.9992 | 0.3870 |
| 177 | PE(35:1) | 0.2408 | 1.27 | 0.1191 | 2.02 | 0.0432 | 0.9992 | 0.4151 |
| 178 | PE(35:2) | 0.3166 | 1.37 | 0.1149 | 2.76 | 0.0059 | 0.9992 | 0.3939 |
| 179 | PE(O-34:1) | 0.1388 | 1.15 | 0.1090 | 1.27 | 0.2030 | 0.9992 | 0.3810 |
| 180 | PE(O-34:2) | -0.0074 | 0.99 | 0.1026 | -0.07 | 0.9427 | 0.9992 | 0.4108 |
| 181 | PE(O-36:2) | 0.0515 | 1.05 | 0.1048 | 0.49 | 0.6232 | 0.9992 | 0.3884 |
| 182 | PE(O-36:3) | 0.0236 | 1.02 | 0.0994 | 0.24 | 0.8123 | 0.9992 | 0.4026 |
| 183 | PE(O-36:4) | 0.0678 | 1.07 | 0.1048 | 0.65 | 0.5177 | 0.9992 | 0.3911 |
| 184 | PE(O-36:5) | 0.0049 | 1.00 | 0.1060 | 0.05 | 0.9630 | 0.9992 | 0.4085 |
| 185 | PE(O-38:4) | 0.0385 | 1.04 | 0.1094 | 0.35 | 0.7247 | 0.9992 | 0.3965 |
| 186 | PE(O-38:5) | 0.0441 | 1.05 | 0.1020 | 0.43 | 0.6652 | 0.9992 | 0.3866 |
| 187 | PE(O-38:6) | 0.1243 | 1.13 | 0.1080 | 1.15 | 0.2494 | 0.9992 | 0.3631 |
| 188 | PE(O-40:5) | 0.0679 | 1.07 | 0.1081 | 0.63 | 0.5299 | 0.9992 | 0.3694 |
| 189 | PE(O-40:6) | 0.0334 | 1.03 | 0.1102 | 0.30 | 0.7621 | 0.9992 | 0.4005 |
| 190 | PE(O-40:7) | 0.0622 | 1.06 | 0.1019 | 0.61 | 0.5419 | 0.9992 | 0.3796 |
| 191 | PE(P-34:1) | 0.1072 | 1.11 | 0.1101 | 0.97 | 0.3302 | 0.9992 | 0.3805 |
| 192 | PE(P-34:2) | 0.1710 | 1.19 | 0.1063 | 1.61 | 0.1076 | 0.9992 | 0.3427 |
| 193 | PE(P-36:4) | 0.2045 | 1.23 | 0.1105 | 1.85 | 0.0642 | 0.9992 | 0.3477 |
| 194 | PE(P-38:6) | 0.2723 | 1.31 | 0.1048 | 2.60 | 0.0094 | 0.9992 | 0.3725 |
| 195 | PE(P-36:1) | 0.1085 | 1.11 | 0.1107 | 0.98 | 0.3269 | 0.9992 | 0.3838 |
| 196 | PE(P-36:2) | 0.1019 | 1.11 | 0.1073 | 0.95 | 0.3423 | 0.9992 | 0.3865 |
| 197 | PE(P-38:4) | 0.0985 | 1.10 | 0.1115 | 0.88 | 0.3771 | 0.9992 | 0.4064 |
| 198 | PE(P-40:5) | 0.0409 | 1.04 | 0.1096 | 0.37 | 0.7091 | 0.9992 | 0.3953 |
| 199 | PE(P-40:6) | 0.1108 | 1.12 | 0.1048 | 1.06 | 0.2904 | 0.9992 | 0.4065 |
| 200 | PG(34:1) | 0.0748 | 1.08 | 0.1225 | 0.61 | 0.5416 | 0.9992 | 0.4081 |
| 201 | PG(34:2) | 0.0514 | 1.05 | 0.1102 | 0.47 | 0.6408 | 0.9992 | 0.3988 |
| 202 | PG(36:1) | 0.2777 | 1.32 | 0.1437 | 1.93 | 0.0534 | 0.9992 | 0.4180 |
| 203 | PG(36:2) | 0.2340 | 1.26 | 0.1336 | 1.75 | 0.0798 | 0.9992 | 0.4224 |
| 204 | PS(36:1) | 0.1512 | 1.16 | 0.1066 | 1.42 | 0.1561 | 0.9992 | 0.3962 |
| 205 | PS(36:2) | 0.1032 | 1.11 | 0.1061 | 0.97 | 0.3304 | 0.9992 | 0.3971 |
| 206 | PS(38:3) | 0.1728 | 1.19 | 0.1046 | 1.65 | 0.0984 | 0.9992 | 0.3953 |
| 207 | PS(38:4) | 0.1000 | 1.11 | 0.1052 | 0.95 | 0.3416 | 0.9992 | 0.3973 |
| 208 | PS(38:5) | 0.0517 | 1.05 | 0.1059 | 0.49 | 0.6251 | 0.9992 | 0.4031 |
| 209 | PS(40:5) | 0.1715 | 1.19 | 0.1016 | 1.69 | 0.0913 | 0.9992 | 0.4070 |
| 210 | PS(40:6) | 0.2630 | 1.30 | 0.1062 | 2.48 | 0.0133 | 0.9992 | 0.3450 |
| 211 | PI(32:1) | 0.2646 | 1.30 | 0.1187 | 2.23 | 0.0258 | 0.9992 | 0.4149 |
| 212 | PI(32:0) | 0.2610 | 1.30 | 0.1177 | 2.22 | 0.0266 | 0.9992 | 0.3966 |
| 213 | PI(34:1) | 0.2798 | 1.32 | 0.1211 | 2.31 | 0.0208 | 0.9992 | 0.4334 |
| 214 | PI(34:0) | 0.2258 | 1.25 | 0.1182 | 1.91 | 0.0561 | 0.9992 | 0.3943 |
| 215 | PI(36:4) | 0.1363 | 1.15 | 0.1301 | 1.05 | 0.2948 | 0.9992 | 0.4089 |
| 216 | PI(36:3) | 0.0481 | 1.05 | 0.1188 | 0.40 | 0.6857 | 0.9992 | 0.4000 |
| 217 | PI(36:2) | 0.0597 | 1.06 | 0.1187 | 0.50 | 0.6149 | 0.9992 | 0.3986 |
| 218 | PI(36:1) | 0.1174 | 1.12 | 0.1167 | 1.01 | 0.3144 | 0.9992 | 0.3931 |
| 219 | PI(36:0) | 0.3212 | 1.38 | 0.1128 | 2.85 | 0.0044 | 0.9992 | 0.3268 |
| 220 | PI(38:6) | 0.1021 | 1.11 | 0.1145 | 0.89 | 0.3723 | 0.9992 | 0.4024 |
| 221 | PI(38:5) | -0.1997 | 0.82 | 0.1172 | -1.70 | 0.0884 | 0.9992 | 0.4306 |
| 222 | PI(38:4) | -0.1039 | 0.90 | 0.1295 | -0.80 | 0.4227 | 0.9992 | 0.3993 |
| 223 | PI(38:3) | 0.1054 | 1.11 | 0.1226 | 0.86 | 0.3897 | 0.9992 | 0.4309 |
| 224 | PI(38:2) | -0.0127 | 0.99 | 0.1201 | -0.11 | 0.9158 | 0.9992 | 0.4050 |
| 225 | PI(40:6) | 0.1746 | 1.19 | 0.1126 | 1.55 | 0.1211 | 0.9992 | 0.3931 |
| 226 | PI(40:5) | 0.1126 | 1.12 | 0.1129 | 1.00 | 0.3188 | 0.9992 | 0.3936 |
| 227 | PI(40:4) | 0.1353 | 1.14 | 0.1287 | 1.05 | 0.2929 | 0.9992 | 0.4302 |
| 228 | Cholesterol | 0.1793 | 1.20 | 0.1271 | 1.41 | 0.1583 | 0.9992 | 0.3930 |
| 229 | CE(14:0) | 0.2455 | 1.28 | 0.1345 | 1.83 | 0.0680 | 0.9992 | 0.3864 |
| 230 | CE(15:0) | 0.2368 | 1.27 | 0.1092 | 2.17 | 0.0301 | 0.9992 | 0.3437 |
| 231 | CE(16:0) | 0.2695 | 1.31 | 0.1125 | 2.40 | 0.0166 | 0.9992 | 0.4034 |
| 232 | CE(16:1) | 0.2518 | 1.29 | 0.1291 | 1.95 | 0.0512 | 0.9992 | 0.4612 |
| 233 | CE(16:2) | 0.3717 | 1.45 | 0.1442 | 2.58 | 0.0100 | 0.9992 | 0.4376 |
| 234 | CE(17:0) | 0.0581 | 1.06 | 0.1051 | 0.55 | 0.5803 | 0.9992 | 0.3895 |
| 235 | CE(17:1) | 0.0570 | 1.06 | 0.1064 | 0.54 | 0.5924 | 0.9992 | 0.4055 |
| 236 | CE(18:0) | 0.1566 | 1.17 | 0.1217 | 1.29 | 0.1983 | 0.9992 | 0.3806 |
| 237 | CE(18:1) | 0.2621 | 1.30 | 0.1166 | 2.25 | 0.0246 | 0.9992 | 0.5068 |
| 238 | CE(18:2) | 0.3091 | 1.36 | 0.1357 | 2.28 | 0.0227 | 0.9992 | 0.5227 |
| 239 | CE(18:3) | 0.3333 | 1.40 | 0.1496 | 2.23 | 0.0259 | 0.9992 | 0.4581 |
| 240 | CE(20:1) | 0.0875 | 1.09 | 0.1139 | 0.77 | 0.4426 | 0.9992 | 0.4101 |
| 241 | CE(20:2) | 0.1254 | 1.13 | 0.1080 | 1.16 | 0.2458 | 0.9992 | 0.4515 |
| 242 | CE(20:3) | 0.3903 | 1.48 | 0.1324 | 2.95 | 0.0032 | 0.9590 | 0.4839 |
| 243 | CE(20:4) | 0.2380 | 1.27 | 0.1396 | 1.70 | 0.0883 | 0.9992 | 0.4731 |
| 244 | CE(20:5) | 0.2432 | 1.28 | 0.1333 | 1.82 | 0.0681 | 0.9992 | 0.4536 |
| 245 | CE(22:0) | 0.1094 | 1.12 | 0.1304 | 0.84 | 0.4011 | 0.9992 | 0.3853 |
| 246 | CE(22:1) | -0.0234 | 0.98 | 0.1158 | -0.20 | 0.8396 | 0.9992 | 0.4095 |
| 247 | CE(22:4) | 0.0457 | 1.05 | 0.1086 | 0.42 | 0.6741 | 0.9992 | 0.4219 |
| 248 | CE(22:5) | 0.1338 | 1.14 | 0.1249 | 1.07 | 0.2843 | 0.9992 | 0.4438 |
| 249 | CE(22:6) | 0.2379 | 1.27 | 0.1305 | 1.82 | 0.0682 | 0.9992 | 0.4753 |
| 250 | CE(24:0) | 0.1756 | 1.19 | 0.1349 | 1.30 | 0.1931 | 0.9992 | 0.3866 |
| 251 | CE(24:1) | -0.0001 | 1.00 | 0.1247 | 0.00 | 0.9992 | 0.9992 | 0.4094 |
| 252 | CE(24:4) | 0.1978 | 1.22 | 0.1020 | 1.94 | 0.0525 | 0.9992 | 0.4564 |
| 253 | CE(24:5) | 0.3070 | 1.36 | 0.1091 | 2.81 | 0.0049 | 0.9992 | 0.4083 |
| 254 | CE(24:6) | 0.2363 | 1.27 | 0.1147 | 2.06 | 0.0395 | 0.9992 | 0.4543 |
| 255 | DG(14:0_16:0) | 0.2283 | 1.26 | 0.1435 | 1.59 | 0.1117 | 0.9992 | 0.3271 |
| 256 | DG(14:0_18:1) | 0.1537 | 1.17 | 0.1512 | 1.02 | 0.3094 | 0.9992 | 0.3636 |
| 257 | DG(14:0_18:2) | 0.1399 | 1.15 | 0.1471 | 0.95 | 0.3413 | 0.9992 | 0.3702 |
| 258 | DG(16:0_16:0) | 0.4423 | 1.56 | 0.1580 | 2.80 | 0.0051 | 0.9992 | 0.3102 |
| 259 | DG(16:0_18:0) | 0.5139 | 1.67 | 0.1624 | 3.16 | 0.0016 | 0.4714 | 0.2837 |
| 260 | DG(16:0_18:1) | 0.5169 | 1.68 | 0.1880 | 2.75 | 0.0060 | 0.9992 | 0.4045 |
| 261 | DG(16:0_18:2) | 0.3628 | 1.44 | 0.1746 | 2.08 | 0.0377 | 0.9992 | 0.3629 |
| 262 | DG(16:0_20:3) | 0.3246 | 1.38 | 0.1533 | 2.12 | 0.0342 | 0.9992 | 0.3529 |
| 263 | DG(16:0_20:4) | 0.3872 | 1.47 | 0.1494 | 2.59 | 0.0096 | 0.9992 | 0.3232 |
| 264 | DG(16:0_22:5) | 0.1709 | 1.19 | 0.1459 | 1.17 | 0.2414 | 0.9992 | 0.4056 |
| 265 | DG(16:0_22:6) | 0.2198 | 1.25 | 0.1380 | 1.59 | 0.1113 | 0.9992 | 0.3955 |
| 266 | DG(16:1_18:1) | 0.0531 | 1.05 | 0.1563 | 0.34 | 0.7338 | 0.9992 | 0.4188 |
| 267 | DG(16:1_18:0) | 0.2078 | 1.23 | 0.1544 | 1.35 | 0.1784 | 0.9992 | 0.3862 |
| 268 | DG(18:0_18:1) | 0.4321 | 1.54 | 0.1803 | 2.40 | 0.0165 | 0.9992 | 0.3953 |
| 269 | DG(18:0_18:2) | 0.3357 | 1.40 | 0.1760 | 1.91 | 0.0564 | 0.9992 | 0.4100 |
| 270 | DG(18:0_20:4) | 0.3067 | 1.36 | 0.1464 | 2.09 | 0.0362 | 0.9992 | 0.3405 |
| 271 | DG(18:1_18:1) | 0.2245 | 1.25 | 0.1707 | 1.32 | 0.1884 | 0.9992 | 0.4483 |
| 272 | DG(18:1_18:2) | 0.0642 | 1.07 | 0.1515 | 0.42 | 0.6716 | 0.9992 | 0.4195 |
| 273 | DG(18:1_18:3) | 0.0542 | 1.06 | 0.1421 | 0.38 | 0.7030 | 0.9992 | 0.4028 |
| 274 | DG(18:1_20:3) | 0.2335 | 1.26 | 0.1534 | 1.52 | 0.1279 | 0.9992 | 0.3824 |
| 275 | DG(18:1_20:4) | 0.2432 | 1.28 | 0.1532 | 1.59 | 0.1124 | 0.9992 | 0.4138 |
| 276 | DG(18:2_18:2) | 0.0053 | 1.01 | 0.1268 | 0.04 | 0.9667 | 0.9992 | 0.4094 |
| 277 | TG(14:0_16:0_18:2) | 0.2563 | 1.29 | 0.1465 | 1.75 | 0.0803 | 0.9992 | 0.4021 |
| 278 | TG(14:0_16:1_18:1) | 0.1286 | 1.14 | 0.1439 | 0.89 | 0.3715 | 0.9992 | 0.4023 |
| 279 | TG(14:0_16:1_18:2) | 0.0523 | 1.05 | 0.1419 | 0.37 | 0.7123 | 0.9992 | 0.4032 |
| 280 | TG(14:0_18:0_18:1) | 0.4209 | 1.52 | 0.1505 | 2.80 | 0.0052 | 0.9992 | 0.3528 |
| 281 | TG(14:0_18:2_18:2) | -0.0608 | 0.94 | 0.1333 | -0.46 | 0.6486 | 0.9992 | 0.4241 |
| 282 | TG(14:1_16:0_18:1) | 0.1397 | 1.15 | 0.1418 | 0.99 | 0.3246 | 0.9992 | 0.4070 |
| 283 | TG(14:1_16:1_18:0) | 0.1731 | 1.19 | 0.1386 | 1.25 | 0.2118 | 0.9992 | 0.4230 |
| 284 | TG(14:1_18:0_18:2) | 0.0244 | 1.02 | 0.1404 | 0.17 | 0.8619 | 0.9992 | 0.4103 |
| 285 | TG(14:1_18:1_18:1) | -0.0685 | 0.93 | 0.1532 | -0.45 | 0.6549 | 0.9992 | 0.4087 |
| 286 | TG(15:0_16:0_18:1) | 0.3465 | 1.41 | 0.1469 | 2.36 | 0.0183 | 0.9992 | 0.3480 |
| 287 | TG(15:0_18:1_18:1) | 0.1702 | 1.19 | 0.1415 | 1.20 | 0.2290 | 0.9992 | 0.4355 |
| 288 | TG(16:0_16:0_16:0) | 0.5240 | 1.69 | 0.1408 | 3.72 | 0.0002 | 0.0625 | 0.3410 |
| 289 | TG(16:0_16:0_18:0) | 0.5949 | 1.81 | 0.1520 | 3.91 | 0.0001 | 0.0287 | 0.3554 |
| 290 | TG(16:0_16:0_18:1) | 0.5926 | 1.81 | 0.1637 | 3.62 | 0.0003 | 0.0914 | 0.4343 |
| 291 | TG(16:0_16:0_18:2) | 0.6123 | 1.84 | 0.1675 | 3.66 | 0.0003 | 0.0804 | 0.3747 |
| 292 | TG(16:0_16:1_18:1) | 0.2594 | 1.30 | 0.1556 | 1.67 | 0.0955 | 0.9992 | 0.4582 |
| 293 | TG(16:0_18:0_18:1) | 0.7186 | 2.05 | 0.1706 | 4.21 | 0.0000 | 0.0080 | 0.4310 |
| 294 | TG(16:0_18:1_18:1) | 0.4679 | 1.60 | 0.1749 | 2.67 | 0.0075 | 0.9992 | 0.5252 |
| 295 | TG(16:0_18:1_18:2) | 0.2514 | 1.29 | 0.1548 | 1.62 | 0.1044 | 0.9992 | 0.4492 |
| 296 | TG(16:0_18:2_18:2) | 0.0179 | 1.02 | 0.1321 | 0.14 | 0.8920 | 0.9992 | 0.4090 |
| 297 | TG(16:1_16:1_16:1) | -0.0513 | 0.95 | 0.1421 | -0.36 | 0.7183 | 0.9992 | 0.4099 |
| 298 | TG(16:1_16:1_18:0) | 0.3349 | 1.40 | 0.1442 | 2.32 | 0.0202 | 0.9992 | 0.3451 |
| 299 | TG(16:1_16:1_18:1) | 0.0860 | 1.09 | 0.1538 | 0.56 | 0.5760 | 0.9992 | 0.4240 |
| 300 | TG(16:1_18:1_18:1) | -0.0453 | 0.96 | 0.1420 | -0.32 | 0.7495 | 0.9992 | 0.3954 |
| 301 | TG(16:1_18:1_18:2) | -0.1295 | 0.88 | 0.1392 | -0.93 | 0.3520 | 0.9992 | 0.3939 |
| 302 | TG(16:0_16:1_17:0) | 0.3781 | 1.46 | 0.1471 | 2.57 | 0.0102 | 0.9992 | 0.3917 |
| 303 | TG(16:0_17:0_18:0) | 0.3123 | 1.37 | 0.1284 | 2.43 | 0.0150 | 0.9992 | 0.3389 |
| 304 | TG(14:0_17:0_18:1) | 0.3078 | 1.36 | 0.1439 | 2.14 | 0.0324 | 0.9992 | 0.3938 |
| 305 | TG(16:0_17:0_18:1) | 0.5106 | 1.67 | 0.1551 | 3.29 | 0.0010 | 0.3029 | 0.4209 |
| 306 | TG(16:1_17:0_18:1) | 0.1677 | 1.18 | 0.1511 | 1.11 | 0.2670 | 0.9992 | 0.4314 |
| 307 | TG(17:0_18:1_18:1) | -0.0353 | 0.97 | 0.1357 | -0.26 | 0.7949 | 0.9992 | 0.4082 |
| 308 | TG(16:0_17:0_18:2) | 0.2745 | 1.32 | 0.1580 | 1.74 | 0.0822 | 0.9992 | 0.4508 |
| 309 | TG(18:0_18:0_18:1) | 0.4151 | 1.51 | 0.1493 | 2.78 | 0.0054 | 0.9992 | 0.3119 |
| 310 | TG(18:0_18:1_18:1) | 0.5420 | 1.72 | 0.1628 | 3.33 | 0.0009 | 0.2657 | 0.4155 |
| 311 | TG(18:0_18:2_18:2) | 0.1024 | 1.11 | 0.1195 | 0.86 | 0.3913 | 0.9992 | 0.4276 |
| 312 | TG(14:0_16:0_18:1) | 0.3484 | 1.42 | 0.1487 | 2.34 | 0.0191 | 0.9992 | 0.3824 |
| 313 | TG(18:1_18:1_18:1) | 0.1784 | 1.20 | 0.1414 | 1.26 | 0.2070 | 0.9992 | 0.4472 |
| 314 | TG(18:1_18:1_18:2) | -0.0079 | 0.99 | 0.1198 | -0.07 | 0.9474 | 0.9992 | 0.4083 |
| 315 | TG(18:1_18:1_20:4) | 0.2339 | 1.26 | 0.1343 | 1.74 | 0.0816 | 0.9992 | 0.4482 |
| 316 | TG(18:1_18:1_22:6) | 0.1171 | 1.12 | 0.1326 | 0.88 | 0.3772 | 0.9992 | 0.4179 |
| 317 | TG(18:1_18:2_18:2) | -0.0010 | 1.00 | 0.1272 | -0.01 | 0.9938 | 0.9992 | 0.4095 |
| 318 | TG(18:2_18:2_18:2) | -0.0927 | 0.91 | 0.1146 | -0.81 | 0.4185 | 0.9992 | 0.4152 |
| 319 | TG(18:2_18:2_20:4) | -0.0995 | 0.91 | 0.1111 | -0.90 | 0.3706 | 0.9992 | 0.4368 |

β, regression coefficient; RH, relative hazard; SE, standard error; Nom-P, nominal p value; FDR-p, FDR corrected p value; REV, random effects variance

**Supplementary Table S5: Median LRS based on T2D status**

| **T2D status** | **SAFHS Cohort** | | | **AusDiab Cohort*** | | |
| --- | --- | --- | --- | --- | --- | --- |
|  | n | Median LRS | IQR | n | Median LRS | IQR |
| NGT at baseline and during follow-up | 560 | -0.24 | 1.27 | 329 | -0.22 | 0.96 |
| Prediabetes at baseline and during follow-up | 89 | 0.35 | 1.10 | 82 | 0.09 | 0.90 |
| T2D during follow-up | 122 | 0.78 | 1.11 | 233 | 0.25 | 0.93 |
| Cuzick’s nonparametric test for linear trend | Z = 10.33, p <1x10^-22^ | | | Z = 8.31, p<1x10^-22^ | | |

*, represents the optimized score for the AusDiab cohort

**Supplementary Table S6:** Incidence rate ratio for the association of LRS, calibrated on the AusDiab cohort, with future type 2 diabetes in the SAFHS cohort estimated using mixed effects Poisson regression.*

| **Model** | **IRR**** | **95% CI** | **P** |
| --- | --- | --- | --- |
| Unadjusted | 2.58 | 2.06 – 3.24 | <0.001 |
| Adjusted for clinical covariates | 2.39 | 1.76 – 3.26 | <0.001 |
| Adjusted for prediabetes | 2.11 | 1.68 – 2.66 | <0.001 |

*, analyses were conducted using the xtmepoisson command in Stata to account for kinship structure as random effects

**, shows incidence ratio per standard deviation of the LRS

**Supplementary Table S7: Probabilities used for base case and sensitivity analyses.**

| Variable* | SAFHS cohort | | AusDiab cohort | |
| --- | --- | --- | --- | --- |
|  | Base Case | Range | Base Case | Range |
| Pr(HR_1_) | 0.1154 | 0.0928 - 0.1380 | 0.5038 | 0.4655 - 0.5422 |
| Pr(HR_2_) | 0.3294 | 0.2962 - 0.3626 | 0.4946 | 0.4563 - 0.5330 |
| Pr(HR_3_) | 0.0610 | 0.0441 - 0.0779 | 0.3247 | 0.2887 - 0.3606 |
| Pr(MR_3_) | 0.3230 | 0.2900 - 0.3560 | 0.3492 | 0.3126 - 0.3857 |
| Pr(HR_4_) | 0.3294 | 0.2962 - 0.3626 | 0.4946 | 0.4563 - 0.5330 |
| Pr(MR_4_) | 0.0545 | 0.0385 - 0.0705 | 0.1792 | 0.1498 - 0.2086 |
| Pr(HR_5_) | 0.1154 | 0.0928 - 0.1380 | 0.3247 | 0.2887 - 0.3606 |
| Pr(MR_5_) | 0.2685 | 0.2372 - 0.2998 | 0.1700 | 0.1412 - 0.1988 |
| Pr(HR_6_) | 0.2905 | 0.2585 - 0.3225 | 0.4701 | 0.4319 - 0.5084 |
| Pr(MR_6_) | 0.4682 | 0.4330 - 0.5034 | 0.3430 | 0.3066 - 0.3794 |
| Pr(T2D\|HR_1_) | 0.1890 | 0.1077 - 0.2703 | 0.5441 | 0.4903 - 0.5979 |
| Pr(T2D\|HR_2_) | 0.1017 | 0.0645 - 0.1389 | 0.4861 | 0.4316 - 0.5406 |
| Pr(T2D\|HR_3_) | 0.2673 | 0.1408 - 0.3938 | 0.5896 | 0.5234 - 0.6558 |
| Pr(T2D\|MR_3_) | 0.0711 | 0.0392 - 0.1030 | 0.3772 | 0.3143 - 0.4401 |
| Pr(T2D\|HR_4_) | 0.1017 | 0.0645 - 0.1389 | 0.4861 | 0.4316 - 0.5406 |
| Pr(T2D\|MR_4_) | 0.1014 | 0.0101 - 0.1927 | 0.4615 | 0.3712 - 0.5518 |
| Pr(T2D\|HR_5_) | 0.1890 | 0.1077 - 0.2703 | 0.5896 | 0.5234 - 0.6558 |
| Pr(T2D\|MR_5_) | 0.0651 | 0.0315 - 0.0987 | 0.2883 | 0.2040 - 0.3726 |
| Pr(T2D\|HR_6_) | 0.1106 | 0.0695 - 0.1517 | 0.4984 | 0.4425 - 0.5543 |
| Pr(T2D\|MR_6_) | 0.0256 | 0.0093 - 0.0419 | 0.2619 | 0.2043 - 0.3195 |

*, variables are named using the following notation: Pr(), probability; HR, MR and LR, high-, moderate- and low-risk, respectively; subscript number, Strategy identifier (see Fig. 4A for strategy description); |, conditional probability; T2D, incident type 2 diabetes

**Supplementary Table S8: Costs and health utilities at the end of five years.** Estimates are based on the DPP data. *(21, 53)*

|  | Year | | | | | Total (2010$) | Total (2015$) |
| --- | --- | --- | --- | --- | --- | --- | --- |
| **Costs** | 1 | 2 | 3 | 4 | 5 |  |  |
| *Cost of intervention* | | | | | | | |
| Lifestyle intervention | 1825.93 | 887.37 | 914.81 | 173.38 | 126.11 | 3927.6 | 4298.26 |
| Metformin | 583.88 | 294.1 | 298.53 | 301.18 | 137.62 | 1615.31 | 1767.75 |
| Placebo | 86.61 | 50.21 | 46.91 | 220.44 | 62.18 | 466.35 | 510.36 |
| *Cost of medical care (Screen detected diabetes)* | | | | | | | |
| Lifestyle intervention | 5799 | 3389 | 3808 | 3626 | 3328 | 19950 | 21832.75 |
| Metformin | 5350 | 2637 | 3290 | 3055 | 3078 | 17410 | 19053.04 |
| Placebo | 2705 | 3724 | 3638 | 3693 | 3339 | 17099 | 18712.69 |
| *Cost of medical care (Non-diabetic)* | | | | | | | |
| Lifestyle intervention | 1795 | 2258 | 2020 | 2271 | 2040 | 10384 | 11363.97 |
| Metformin | 1935 | 2289 | 2243 | 2329 | 2151 | 10947 | 11980.10 |
| Placebo | 1978 | 2106 | 2431 | 2490 | 2337 | 11342 | 12412.38 |
| *Total Costs (Screen detected diabetes)* | | | | | | | |
| Lifestyle intervention | 7624.93 | 4276.37 | 4722.81 | 3799.38 | 3454.11 | 23877.6 | 26131.01 |
| Metformin | 5933.88 | 2931.1 | 3588.53 | 3356.18 | 3215.62 | 19025.31 | 20820.79 |
| Placebo | 2791.61 | 3774.21 | 3684.91 | 3913.44 | 3401.18 | 17565.35 | 19223.05 |
| *Total costs (Non-diabetic)* | | | | | | | |
| Lifestyle intervention | 3620.93 | 3145.37 | 2934.81 | 2444.38 | 2166.11 | 14311.6 | 15662.23 |
| Metformin | 2518.88 | 2583.1 | 2541.53 | 2630.18 | 2288.62 | 12562.31 | 13747.86 |
| Placebo | 2064.61 | 2156.21 | 2477.91 | 2710.44 | 2399.18 | 11808.35 | 12922.74 |
| **Utility scores** | Year | | | | | Total |  |
|  | 1 | 2 | 3 | 4 | 5 |  |  |
| *Screen detected diabetes* | | | | | | |  |
| Lifestyle intervention | 0.665 | 0.644 | 0.665 | 0.652 | 0.659 | 3.285 |  |
| Metformin | 0.717 | 0.681 | 0.685 | 0.677 | 0.668 | 3.428 |  |
| Placebo | 0.688 | 0.681 | 0.663 | 0.669 | 0.669 | 3.370* |  |
| *Non-diabetic* | | | | | | |  |
| Lifestyle intervention | 0.703 | 0.698 | 0.695 | 0.695 | 0.688 | 3.479 |  |
| Metformin | 0.688 | 0.683 | 0.682 | 0.681 | 0.682 | 3.416 |  |
| Placebo | 0.689 | 0.677 | 0.678 | 0.676 | 0.675 | 3.395 |  |

*, greater QALYs in the placebo group compared to lifestyle intervention may reflect the perceived preferences rather than a better quality of life

**Supplementary Table S9. Summary of the results of base case cost-effectiveness analyses**

| Strategy* | Cost | QALY | IncrCost | IncrQALY | ICER | T2D risk | NNT in |
| --- | --- | --- | --- | --- | --- | --- | --- |
|  | (2015 US$) |  |  |  |  | Reduction** | HR group |
| **SAFHS cohort** | | | | | | | |
| 1 | 13740.39 | 3.39977 | 118.78 | -0.00215 | -55081.68 | 0.2547 | 79.03 |
| 2 | 14365.96 | 3.41577 | 744.35 | 0.01385 | 53739.33 | 0.3933 | 51.46 |
| 3 | 13621.61 | 3.40192 | Reference | Reference | Reference | 0.2103 | 51.46 |
| 4 | 13864.48 | 3.40376 | 242.87 | 0.00184 | 132428.32 | 0.3328 | 60.35 |
| 5 | 14428.75 | 3.41712 | 807.14 | 0.0152 | 53118.45 | 0.4266 | 47.29 |
| 6 | 13735.9 | 3.40345 | 114.29 | 0.00153 | 74852.84 | 0.2994 | 67.25 |
| 7 | 13822.8 | 3.40106 | 201.19 | -0.00086 | -232168.71 | 0.2018 | 100.39 |
| **AusDiab cohort** | | | | | | | |
| 1 | 17368.67 | 3.38375 | 2171.85 | -0.02422 | -89662 | 0.4362 | 6.29 |
| 2 | 16960.11 | 3.3896 | 1763.29 | -0.01837 | -95995.65 | 0.3826 | 13.42 |
| 3 | 15196.82 | 3.40797 | Reference | Reference | Reference | 0.2045 | 13.42 |
| 4 | 17179.95 | 3.39391 | 1983.13 | -0.01406 | -141017.34 | 0.4166 | 6.59 |
| 5 | 17662 | 3.39452 | 2465.18 | -0.01345 | -183255.57 | 0.4529 | 6.06 |
| 6 | 16462.84 | 3.38908 | 1266.02 | -0.01889 | -67001.74 | 0.3463 | 7.92 |
| 7 | 15883.93 | 3.40534 | 687.11 | -0.00263 | -260688.13 | 0.2159 | 13.77 |

*, Details of screening strategies are pictorially shown in Fig. 4A.

QALY, quality-adjusted life years; IncrCost, incremental cost compared to the reference strategy; IncrQALY, incremental QALY compared to the reference strategy; ICER, incremental cost/effectiveness ratio; NNT, number needed to treat defined as 1/absolute risk reduction; HR, high risk

**, fraction of T2D incidence reduced due to screening/prevention strategy
